# Construction of a redox-related gene signature for overall survival prediction and immune infiltration in non-small-cell lung cancer

**DOI:** 10.3389/fmolb.2022.942402

**Published:** 2022-08-16

**Authors:** Ti-wei Miao, De-qing Yang, Li-juan Gao, Jie Yin, Qi Zhu, Jie Liu, Yan-qiu He, Xin Chen

**Affiliations:** ^1^ Department of Integrated Traditional Chinese and Western Medicine, Zigong First People’s Hospital, Zigong, China; ^2^ Department of Integrated Traditional Chinese and Western Medicine, West China Hospital of Sichuan University, Chengdu, China; ^3^ Department of Pharmacy, The Second Affiliated Hospital of Kunming Medical University, Kunming, China; ^4^ Division of Pulmonary Diseases, Department of Respiratory and Critical Care Medicine, West China Hospital of Sichuan University, Chengdu, China; ^5^ School of Automation and Information Engineering, Sichuan University of Science and Engineering, Zigong, China

**Keywords:** gene signature, NSCLC, cancer prognosis, immune infiltration, redox homeostasis

## Abstract

**Background:** An imbalance in the redox homeostasis has been reported in multiple cancers and is associated with a poor prognosis of disease. However, the prognostic value of redox-related genes in non-small-cell lung cancer (NSCLC) remains unclear.

**Methods:** RNA sequencing data, DNA methylation data, mutation, and clinical data of NSCLC patients were downloaded from The Cancer Genome Atlas and Gene Expression Omnibus databases. Redox-related differentially expressed genes (DEGs) were used to construct the prognostic signature using least absolute shrinkage and selection operator (LASSO) regression analysis. Kaplan–Meier survival curve and receiver operator characteristic (ROC) curve analyses were applied to validate the accuracy of the gene signature. Nomogram and calibration plots of the nomogram were constructed to predict prognosis. Pathway analysis was performed using gene set enrichment analysis. The correlations of risk score with tumor stage, immune infiltration, DNA methylation, tumor mutation burden (TMB), and chemotherapy sensitivity were evaluated. The prognostic signature was validated using GSE31210, GSE26939, and GSE68465 datasets. Real-time polymerase chain reaction (PCR) was used to validate dysregulated genes in NSCLC.

**Results:** A prognostic signature was constructed using the LASSO regression analysis and was represented as a risk score. The high-risk group was significantly correlated with worse overall survival (OS) (*p* < 0.001). The area under the ROC curve (AUC) at the 5-year stage was 0.657. The risk score was precisely correlated with the tumor stage and was an independent prognostic factor for NSCLC. The constructed nomogram accurately predicted the OS of patients after 1-, 3-, and 5-year periods. DNA replication, cell cycle, and ECM receptor interaction were the main pathways enriched in the high-risk group. In addition, the high-risk score was correlated with higher TMB, lower methylation levels, increased infiltrating macrophages, activated memory CD4^+^ T cells, and a higher sensitivity to chemotherapy. The signature was validated in GSE31210, GSE26939, and GSE68465 datasets. Real-time PCR validated dysregulated mRNA expression levels in NSCLC.

**Conclusions:** A prognostic redox-related gene signature was successfully established in NSCLC, with potential applications in the clinical setting.

## Introduction

Lung cancer is the leading cause of cancer-related deaths worldwide, with an estimated 1.8 million deaths (18% of the total number of cancer deaths) (Sung et al., 2021). Non-small cell lung cancer (NSCLC) is a major type of lung cancer, accounting for approximately 85% of all lung cancers (Travis et al., 2015a; Travis et al., 2015b). Only 25% of patients with NSCLC are diagnosed during the early stages (Ni et al., 2018). Furthermore, cancer in approximately 80% of the diagnosed patients is already in the metastatic stage (Goldstraw et al., 2016). Despite recent progress in novel medications, such as targeted therapies and immunotherapies for NSCLC (Facchinetti et al., 2016), the 5 years survival rate remains below 15% (Goldstraw et al., 2016). Therefore, an effective biomarker is urgently required to predict the overall survival (OS) of patients diagnosed with NSCLC.

Advancements in the next-generation sequencing technology have identified multiple biomarkers correlated with prognosis, immune infiltration, and therapy in lung cancer, such as adhesion molecule interacting with CXADR antigen 1 (AMICA1) (Feng et al., 2022), chromobox protein homolog 3 (CBX3) (Niu et al., 2022), and lysine acetyltransferase 2B (KAT2B) (Zhou et al., 2022). However, the researchers have demonstrated that the construction of gene signature could predict better prognosis and therapy response. For instance, hypoxia-related gene signature (Chen et al., 2022), pyroptosis-related gene signature (Zhang and Yan, 2021; Li et al., 2022), autophagy-related gene signature (Wu et al., 2021a), and inflammatory response-related signature (Zou et al., 2021) may better predict prognosis, influence immunological state and guide individualized therapy due to its multidimensional parameters.

Oxidative stress is typically caused by the accumulation of reactive oxygen species (ROS) (Li et al., 2021a). ROS production in cells can cause oxidative damage resulting from DNA point mutations (Lee et al., 2002; Ogrunc et al., 2014), disrupted lipid membranes (Halliwell and Chirico, 1993), and altered protein function (LeDoux et al., 1999; Kwon et al., 2004; Gao et al., 2007). Such oxidative damages are countered by antioxidant systems (Handy and Loscalzo, 2012), which scavenge harmful ROS. Normal ROS and antioxidant levels maintain redox homeostasis in healthy cells, while increased ROS levels are observed in cancer cells. These increased levels lead to genomic instability, facilitate cell proliferation, increase motility, and activate oncogenic signaling (Moloney and Cotter, 2018; Forman and Zhang, 2021). ROS can participate in several cancer signaling pathways implicated in tumor growth, metabolism, differentiation, and metastasis (Aggarwal et al., 2019). Importantly, redox imbalance has also been observed in NSCLC (Weng et al., 2018; Zhao et al., 2021a). For example, antioxidant enzymes including superoxide dismutase (SOD), catalase (CAT), and glutathione peroxidase (GPX) are all reduced in NSCLC in comparison with corresponding controls (Zalewska-Ziob et al., 2019), and glutathione (GSH) and its related enzymes that detoxify ROS, are increased in lung cancer (Luengo et al., 2019). Cellular ROS level is usually regulated by nuclear factor-erythroid 2-related factor 2 (NRF2) and its repressor Kelch-like ECH-associated protein 1 (KEAP1) (Zhang et al., 2015). Large-scale genomic studies have revealed that the KEAP1/NRF2 pathway is altered in 23% of lung adenocarcinoma (LUAD) and 34% of lung squamous cell carcinoma (LUSC), approximately (The Cancer Genome Atlas Research Network, 2014). Moreover, the upregulated activity and expression of the NADPH oxidase (NOX) family correlate with tumorigenesis of lung cancer through ROS production (Han et al., 2016). Additionally, cigarette smoking is the most important risk factor for lung cancer, which accounts for nearly two-thirds of all lung cancer cases (Hedyed et al., 2021), and cigarette smoking could induce oxidative stress and mitochondrial damage leading to metabolic reprogramming associated with increased glycolytic flux, contributing to epithelial-mesenchymal transition processes, and cell migration, therefore, promoting tumor progression (Di Vincenzo et al., 2021). However, there are no systematic studies on the role of redox-related genes in NSCLC. Thus, a better understanding of the molecular composition of these genes and their roles in NSCLC is necessary for its accurate prognosis and treatment.

In our study, a redox-related gene signature was established using the least absolute shrinkage and selection operator (LASSO) regression analysis to predict the OS, immune infiltration, and evaluate therapy response in patients diagnosed with NSCLC.

## Materials and methods

### Data preprocessing and identification of DEGs

RNA sequencing data, DNA methylation data, mutation data, and clinical information of patients with NSCLC were downloaded from the cancer genome atlas (TCGA) (https://www.cancer.gov/about-nci/organization/ccg/research/structural-genomics/tcga). A total of 1,037 cancer and 108 normal samples with sequencing data, 807 cancer and 71 normal samples with DNA methylation data, 1,059 tumor samples with mutation data, and 1,027 tumor samples with clinical data were obtained. The GSE31210, GSE68465, and GSE26939 datasets were downloaded from gene expression omnibus (GEO) database (https://www.ncbi.nlm.nih.gov/geo/), which included 226, 442, 116 patients diagnosed with NSCLC, respectively. GSE31210 was from GPL570 [HG-U133_Plus_2] Affymetrix Human Genome U133 Plus 2.0 Array, GSE68465 from GPL96 [HG-U133A] Affymetrix Human Genome U133A Array, and GSE26939 from GPL9053 Agilent-UNC-custom-4X44K. Patients with incomplete follow-up were excluded from the analysis. Therefore, TCGA cohort, GSE31210, GSE68465, and GSE26939 datasets included 978, 226, 442, and 114 patients with complete follow-up information, respectively. Baseline characteristics of patients with NSCLC are listed in Table 1. In TCGA database, 767 and 754 patients with complete follow-up data have mutation data, and methylation data, respectively. The sequencing data from TCGA database and GEO database were analyzed as the training cohort and test cohort, respectively. The redox-related gene sets were obtained from GSEA-MSigDB database (https://www.gsea-msigdb.org/gsea/msigdb), and two redox-related gene sets, namely “GO_CELL_REDOX_HOMEOSTASIS” and “GO_RESPONSE_TO_REDOX_STATE”, were downloaded for further analysis. A total of 4,087 redox-related genes were included in the current study (Supplementary Table S1) (Cook et al., 2011; Gerstberger et al., 2014). The redox-related differentially expressed genes (DEGs) were identified in TCGA cohort using the “limma package” in R programming language (Version 4.0.2) based on |log2 fold change (FC)| ≥ 2 and adjusted *p-value* < 0.05.

**TABLE 1:** Clinical features of patients from TCGA and GEO databases.

Clinical characteristic	TCGA cohorts (978)	GSE31210 (226)	GSE26939 (114)	GSE68465 (442)
**Platform**	TCGA	GPL570	GPL9053	GPL96
**Age (years)**				
>=65	590 (60.33%)	62 (0.27%)	61 (53.51%)	228 (51.58%)
<65	388 (39.67%)	164 (72.57%)	53(46.49%)	214 (48.42%)
unknown	0	0	0	0
**Gender**				
Male	585 (59.82%)	105 (46.46%)	52 (46.61%)	223 (50.45%)
Female	393 (40.185)	121 (53.54)	62 (54.39%)	219 (49.55%)
unknown	0	0	0	0
**T classification**				
T1-T2	819 (83.74%)	226 (100%)	/	401 (90.72%)
T3-T4	156 (15.95%)	0	/	39 (8.82%)
unknown	3 (0.31%)	0	/	2 (0.45%)
**N classification**				
N0	629 (64.31%)	/	/	299 (67.65%)
N1-N3	333 (34.05%)	/	/	140 (31.67%)
unknown	16 (1.64%)	/	/	3 (0.68%)
**M classification**				
M0	722 (73.82%)	/	/	/
M1	31 (3.17%)	/	/	/
unknown	225 (23.01%)	/	/	/
**UICC stage**				
Stage I-II	772 (78.94%)	226 (100%)	81 (71.05%)	/
Stage III-IV	194 (19.84%)	0	20 (17.54%)	/
unknown	12 (1.23%)	0	13 (11.40%)	/

### Construction and validation of the prognostic signature

Univariate Cox regression analysis was performed to identify prognostic genes using redox-related DEGs with *p* < 0.05. Subsequently, a prognostic signature was constructed using LASSO regression analysis following a linear combination of gene expression values multiplied by a regression coefficient (*β*). A formula was as follows: risk score = ∑ (coefficient i × expression of gene i). All patients could obtain a risk score using the formula and be divided into the low-risk or high-risk groups according to the median risk score. Kaplan–Meier survival curve was calculated between the two risk groups and was compared using the log-rank test. Receiver operator characteristic (ROC) curve analysis was used to assess the predictive accuracy of the prognostic signature, and area under the ROC curve (AUC) values with 95% confidence intervals (CIs) were calculated. The prognostic signature was validated using GSE31210, GSE68465, and GSE26939 datasets.

### Correlation of risk score with clinical characteristics

The risk score in different age groups (≥65 and <65 years), biological sexes (female and male), Union for International Cancer Control (UICC) stages (stages _I_, _II_, _III_, and _IV_), T stages (T_1_, T_2_, T_3_, and T_4_), N stages (N_0_, N_1_, N_2_, and N_3_), and M stages (M_0_ and M_1_) were compared using R programming language in TCGA cohort. The clinical characteristics including age, gender, smoking history, UICC stage, and risk score were applied to identify independent prognostic factors contributing to NSCLC using univariate and multivariate Cox regression analyses.

### Nomogram prediction model

A Nomogram prediction model was constructed to predict the 1-, 3-, and 5-years OS rates using independent prognostic factors with the “rms package” in R programming language, and calibration plots of the nomogram were applied to compare the nomogram-predicted probability of OS and the actual OS.

### Gene set enrichment analysis

Gene set enrichment analysis (GSEA) is a computational method that determines whether *a priori* defined set of genes shows statistically significant and concordant differences between two biological states (Subramanian et al., 2005). The high- and low-risk groups were defined according to the median risk score in NSCLC. GSEA (version 4.0.3) was applied to perform Kyoto Encyclopedia of Genes and Genomes (KEGG) pathway analysis between the high- and low-risk expression groups.

### Analysis of DNA methylation and mutation

The DNA methylation data were normalized as the beta value in TCGA database and the values of DNA methylation expression were excluded when their value was missing or zero. The analysis of DNA methylation was performed using the patients with DNA methylation data and complete follow-up data. DNA methylation levels of prognostic genes were compared between the high- and low-risk groups. The mutation data of LUAD and LUSC were allocated in two different files in TCGA database, thus gene mutation frequency of the two risk groups was calculated in LUAD, and LUSC, respectively, using the “maftools package” in R programming language. The correlation of risk score with TMB was evaluated in all NSCLC using the “ggpubr package” and “reshape2 package” in R programming language, and statistical analysis was performed using the Mann–Whitney test and Spearman rank correlation analysis. Kaplan–Meier survival curve was calculated between the high-risk score + high TMB group and the low-risk score + low TMB group and was compared using the log-rank test.

### Analysis of immune infiltration

The gene expression matrix of NSCLC firstly converted tumor micro-environment score matrix using “commonGenes.gct,” “estimateScore.gct,” and “uniq.symbol.txt” in R programming language, and then immune-infiltration profiles were evaluated between the two risk groups and displayed in a violin plot by using the “vioplot” package. The correlation of the risk score with immune infiltration was calculated and displayed using Spearman rank correlation analysis with “limma package,” “reshape2 package,” “tidyverse package,” “ggplot2 package,” “ggpubr package,” and “ggExtra package” in R programming language.

### Analysis of chemotherapy sensitivity

To assess the clinical therapeutic value of prognostic signature, the half inhibitory concentration (IC50) of different chemotherapy drugs was evaluated using all patients in prognostic signature with “pRRophetic packages” in R programming language. Nine chemotherapy drugs, namely bortezomib (Wang et al., 2022a), dasatinib (Kim et al., 2021; Redin et al., 2021), docetaxel (Xiao et al., 2022a; Matsumoto et al., 2022), midostaurin (Ctortecka et al., 2018), paclitaxel (Duan et al., 2022), parthenolide (Li et al., 2020a), pazopanib (Tanimoto et al., 2018), shikonin (Pan et al., 2021), and thapsigargin (Jaskulska et al., 2020) were collected from the previous literature, which has proven to have the potential of anti-tumor in lung cancer.

### Real-time polymerase chain reaction validation

Sixteen paired lung tissues were obtained from patients with NSCLC in Zigong First People’s Hospital, and the clinical information was summarized in Table 2. Total RNA was extracted by using the E. Z.N.A. HP Total RNA Kit (Omega, GA, United States ). Complementary DNA (cDNA) was synthesized by using the PrimeScript™ RT reagent kit (Takara, Japan). Real-time PCR was performed by using iQ ™ SYBR Green Supermix (Bio-Rad). Levels of gene relative expression were normalized to β-actin Ct value, using a 2^−ΔΔCt^ relative quantification method. The primer sequences used were listed in Table 3.

**TABLE 2:** Clinical characteristic of 16 paired lung tissue from patients with NSCLC.

Clinical characteristic	Patients (16)
Age, years	70.5 (66–74.5)
Male, n (%)	16 (100%)
Smokng status, n (%)	16 (100%)
UICC stage I-II, n (%)	5 (31.25%)

**TABLE 3:** The primer sequences.

Gene symbol	Primer symbol	Primer sequences
CDC25C	CDC25C-forward	CTGCCACTCAGCTTACCACT
	CDC25C-reverse	AAGCTGTGCTGGGCTACATT
GRIA1	GRIA1-forward	TGCTTGGTAGATGGTGCTTGAT
	GRIA1-reverse	CTGTGAGTTGCGACAAAGCAA
CHEK2	CHEK2-forward	CTGCAGGTTTAGCGCCACTC
	CHEK2-reverse	TCAGCAGTGGTTCATCAAAGC
KLK6	KLK6-forward	ACACGCTGTAGCTGTCTCCC
	KLK6-reverse	AGCAATCAGACTCAGCACCA
S100P	S100P-forward	TCAAGGTGCTGATGGAGAAGG
	S100P-reverse	TTGCAGCCACGAACACTATGA
COL1A1	COL1A1-forward	GGCTCCTGCTCCTCTTAGC
	COL1A1-reverse	CACACGTCTCGGTCATGGTA
CAV1	CAV1-forward	ACAGGGCAACATCTACAAGCC
	CAV1-reverse	ATGCCGTCAAAACTGTGTGT
SCN1A	SCN1A-forward	AGTGTAGGAGACACACTGCT
	SCN1A-reverse	GCACTGTTTGCTCCATCTTGTC
CYP24A1	CYP24A1-forward	GCAGCCTAGTGCAGATTTCC
	CYP24A1-reverse	GCACTTGGGGATTACGGGAT
GPR37	GPR37-forward	TGTCACCCCAGTCCTCCTTT
	GPR37-reverse	GAGTTCGAGTTCCGTGGTGT
SLC7A5	SLC7A5-forward	GTGACGCTGGTGTACGTGCT
	SLC7A5-reverse	GGGTGGATCATGGAGAGGAT
β-actin	β-actin-forward	CCACGAAACTACCTTCAACTCC
	β-actin-reverse	GTGATCTCCTTCTGCATCCTGT

### Statistical analysis

All statistical analyses were performed using R software (version 4.0.2) or GraphPad Prism (version 7.00). Comparisons between the two groups were quantified using Student’s *t*-test for parametric data or the Mann–Whitney test for nonparametric data. Statistical significance among multiple groups was tested using the Kruskal–Wallis test or analysis of variance (ANOVA). Statistical significance was set at values of *p < 0.05*.

## Results

### Identification of DEGs

**FIGURE 1:**
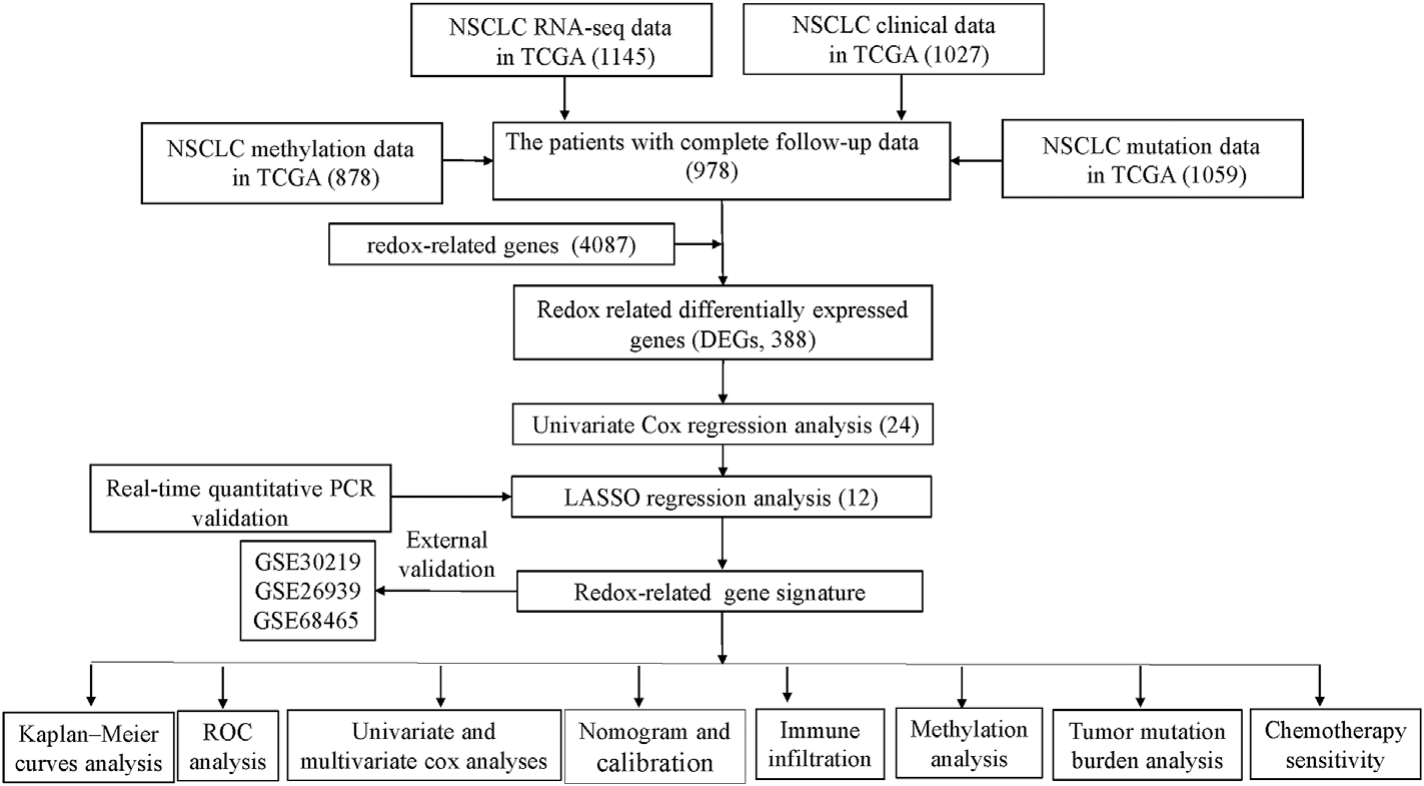
Schematic flowchart representing the procedure followed for establishing the gene signature, and the main findings of the study. Numbers within parenthesis indicate the size of the sample obtained. NSCLC, Non-small cell lung cancer; PCR, polymerase chain reaction; LASSO, least absolute shrinkage, and selection operator; ROC: receiver operator characteristic; TCGA, The Cancer Genome Atlas.

The flowchart depicting the procedure followed in the study is shown in Figure 1. A total of 388 DEGs (279 up-regulated and 109 down-regulated genes) were identified in TCGA cohort when cancer tissues compared with control tissues.

### Construction of a prognostic signature in the TCGA cohort

**FIGURE 2:**
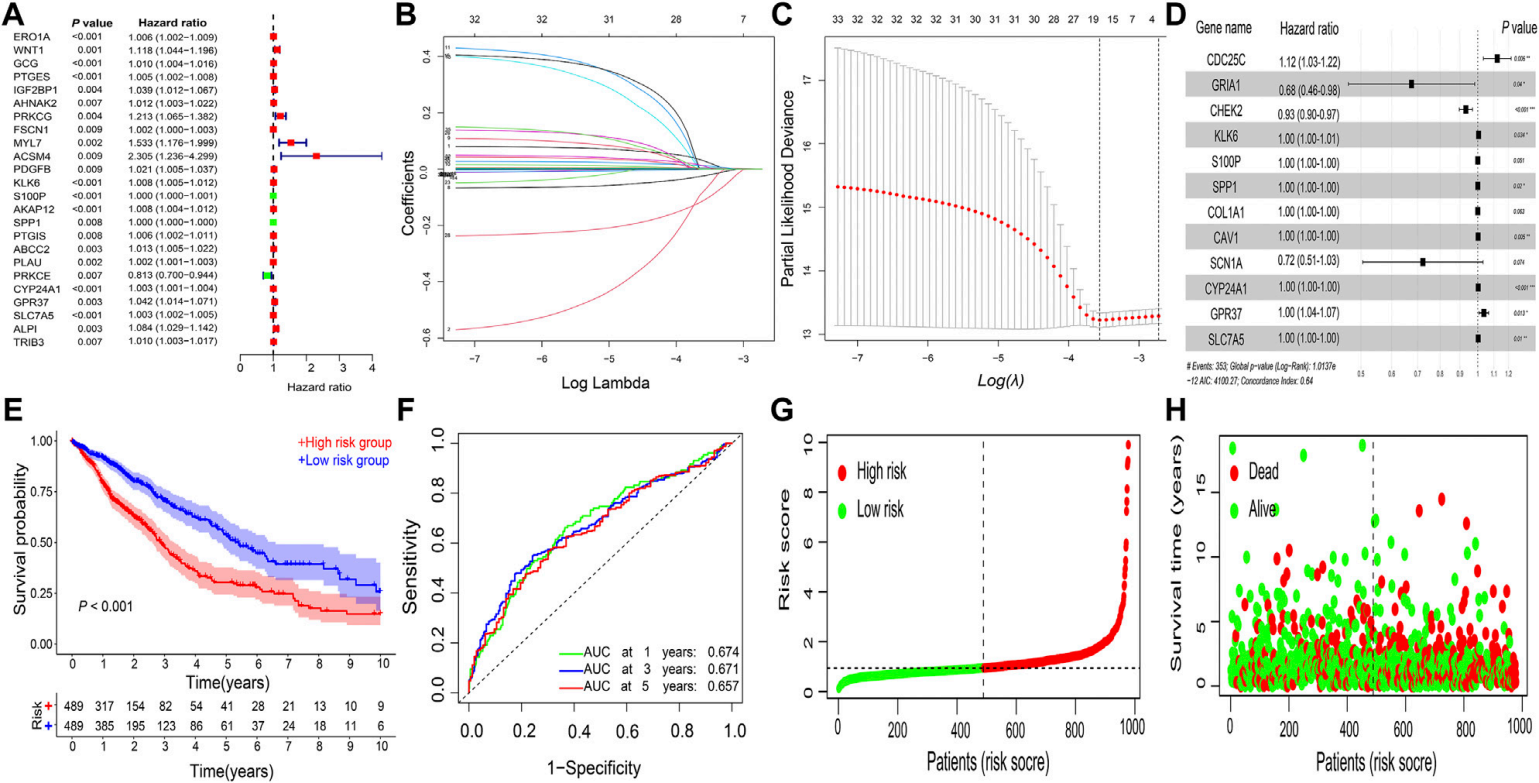
Construction of gene signature. Gene signature constructed using **(A)** univariate Cox regression analysis and **(B–D)** LASSO regression analysis in NSCLC. **(E)** Kaplan–Meier survival analysis. **(F)** ROC curve analysis. **(G)** risk score distribution. **(H)** survival status of the prognostic gene signature. LASSO, least absolute shrinkage and selection operator; NSCLC, non-small cell lung cancer; ROC, receiver operating characteristic; AUC, area under the ROC curve.

Among the 388 DEGs, a total of 24 genes were correlated with prognosis using univariate Cox regression analysis (Figure 2A). Furthermore, a prognostic signature comprising 12 genes (CDC25C, GRIA1, CHEK2, KLK6, S100P, SPP1, COL1A1, CAV1, SCN1A, CYP24A1, GPR37, and SLC7A5) was constructed using the LASSO regression analysis (Figures 2B–D). The risk score for each patient was calculated as follows:

$$\begin{array}{l} \text{risk}\;\text{core}=\left(0.1159\times \text{CDC}25\text{Cexp}\right)+\left(\left[-0.3928\right]\times \text{GRIA}1\text{exp}\right)+\left(\left[-0.0703\right]\times \text{CHEK}2\text{exp}\right)+\left(0.005\times \text{KLK}6\text{exp}\right)+\left(0.0002\times \text{S}100\text{Pexp}\right)+\\ \left(0.0002\times \text{SPP}1\text{exp}\right)+\left(0.0002\times \text{COL}1\text{A}1\text{exp}\right)+\left(0.0021\times \;\text{CAV}1\text{exp}\right)+\left(\left[-0.3256\right]\times \;\text{CN}1\text{Aexp}\right)+\left(0.0026\times \text{CYP}24\text{A}1\text{exp}\right)+\\ \left(0.0361\times \text{GPR}37\text{exp}\right)+\left(0.0026\times SLC7A5\text{exp}\right) \end{array}$$

Then, based on the median risk score of 0.9403, 978 patients were divided into the high- and low-risk subgroups (Figure 2G). Kaplan–Meier survival curve showed that the high-risk group had a worse OS than the low-risk group (*p* < 0.001; Figure 2E), and the AUC values for OS at the 1-, 3-, 5-year periods were 0.674, 0.671, and 0.657, respectively (Figure 2F). Patients in the high-risk group had higher mortality rates than those in the low-risk group (Figure 2H).

### Validation of the prognostic signature in GEO datasets

**FIGURE 3:**
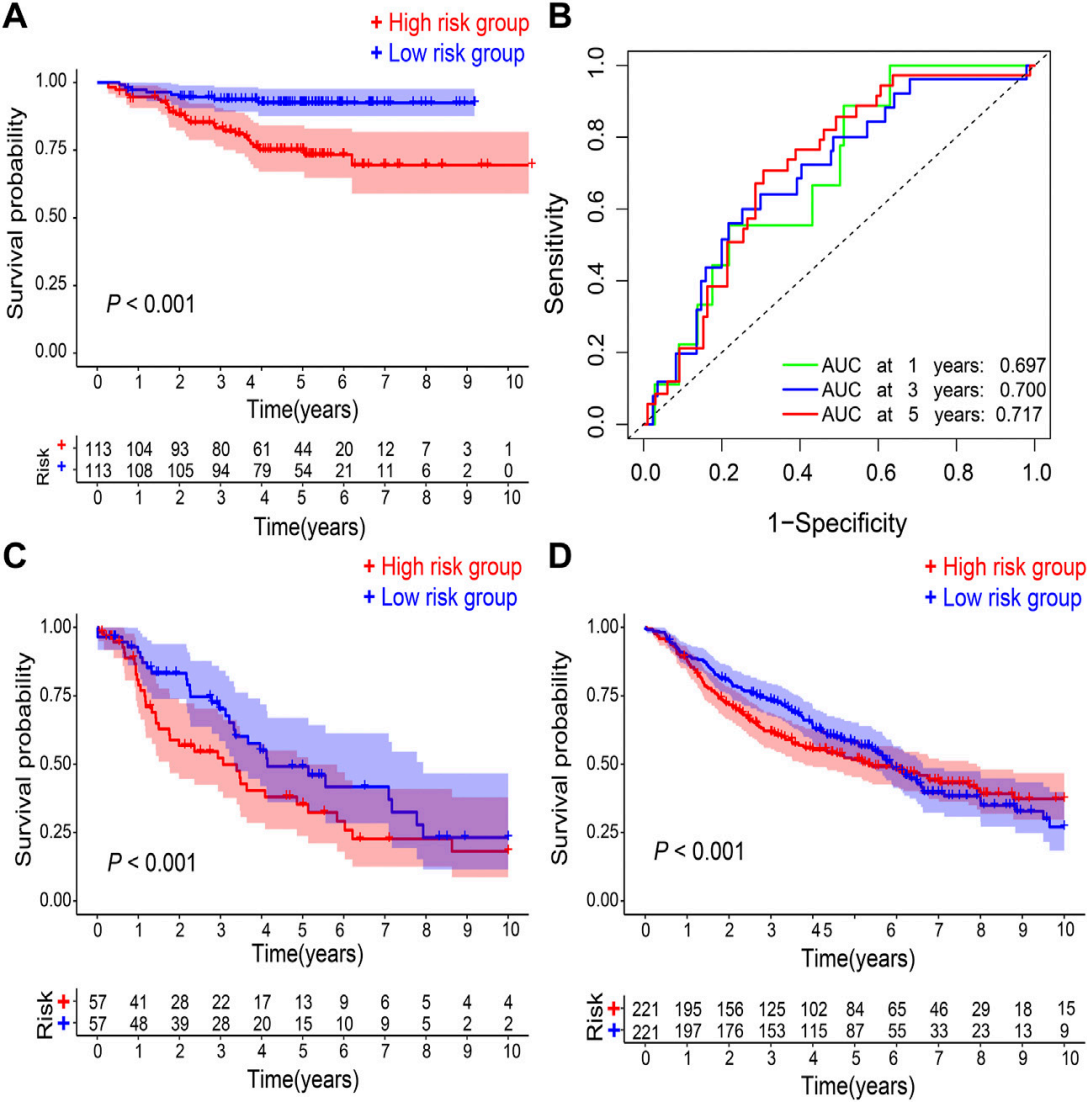
Validation of gene signature. **(A)** Kaplan–Meier survival analysis and **(B)** ROC curve analysis in GSE31210. Kaplan–Meier survival analysis in **(C)** GSE26939 and **(D)** GSE68465, respectively. ROC, receiver operating characteristic. AUC, an area under the ROC curve.

In GSE31210 dataset, compared with the low-risk group, the high-risk group had a worse OS (*p* < 0.001; Figure 3A), and the 1-, 3-, 5-years AUC was 0.687, 0.700, and 0.717, respectively (Figure 3B). Additionally, patients in the high-risk group have also a worse OS in comparison with the low-risk group in both GSE68465 and GSE26939 datasets (*p* < 0.001; Figures 3C,D).

### Correlation of risk score with clinical characteristics

**FIGURE 4:**
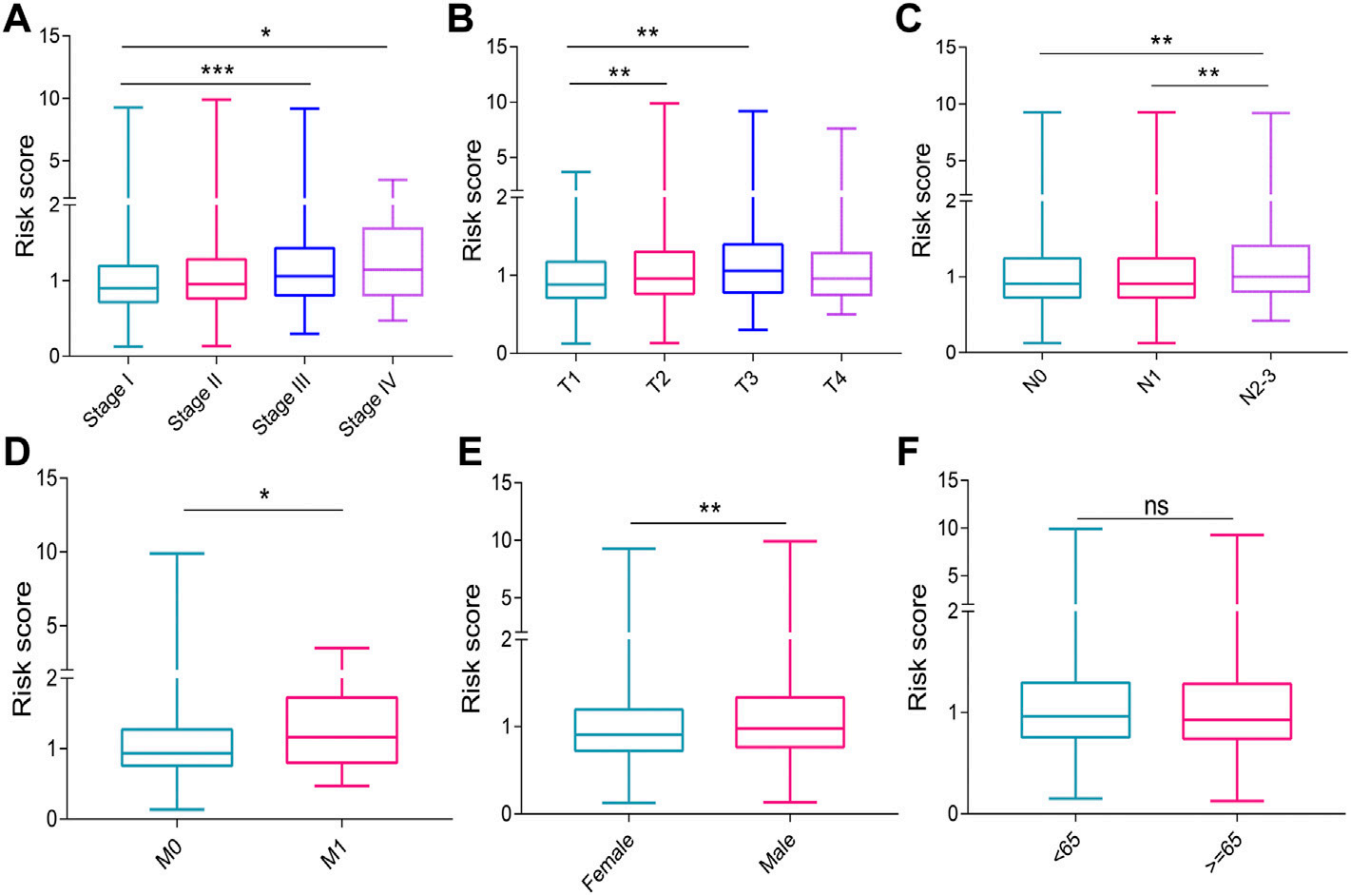
Graphs showing the correlation of risk score with clinical characteristics. **(A)** UICC stages. **(B)** T stages. **(C)** N stages. **(D)** M stages. **(E)** genders. **(F)** ages. UICC, Union for International Cancer Control. ns, no significance. *p < 0.05; **p < 0.01; ***p < 0.001.

The risk score in patients with stage _II_ (*p* = 0.066), stage _III_ (*p* < 0.001), and stage _IV_ (*p* = 0.010) cancer was higher than that in patients with stage _I_ cancer. (Figure 4A). Patients with T_2_ (*p* = 0.004) and T_3_ (*p* = 0.003) had higher risk scores than those with T_1_ (Figure 4B). The risk scores of patients with N_2-3_ were higher than those of patients with N_0_ (*p* = 0.002) and N_1_ (*p* = 0.002) (Figure 4C). The patients with M_1_ had a higher risk score than those with M_0_ (*p* = 0.036, Figure 4D). The male patients have a higher risk score compared with female patients (*p* = 0.002 Figure 4E). The risk score did not differ significantly between the different age groups (Figure 4F).

### Risk score as an independent NSCLC prognostic factor

**FIGURE 5:**
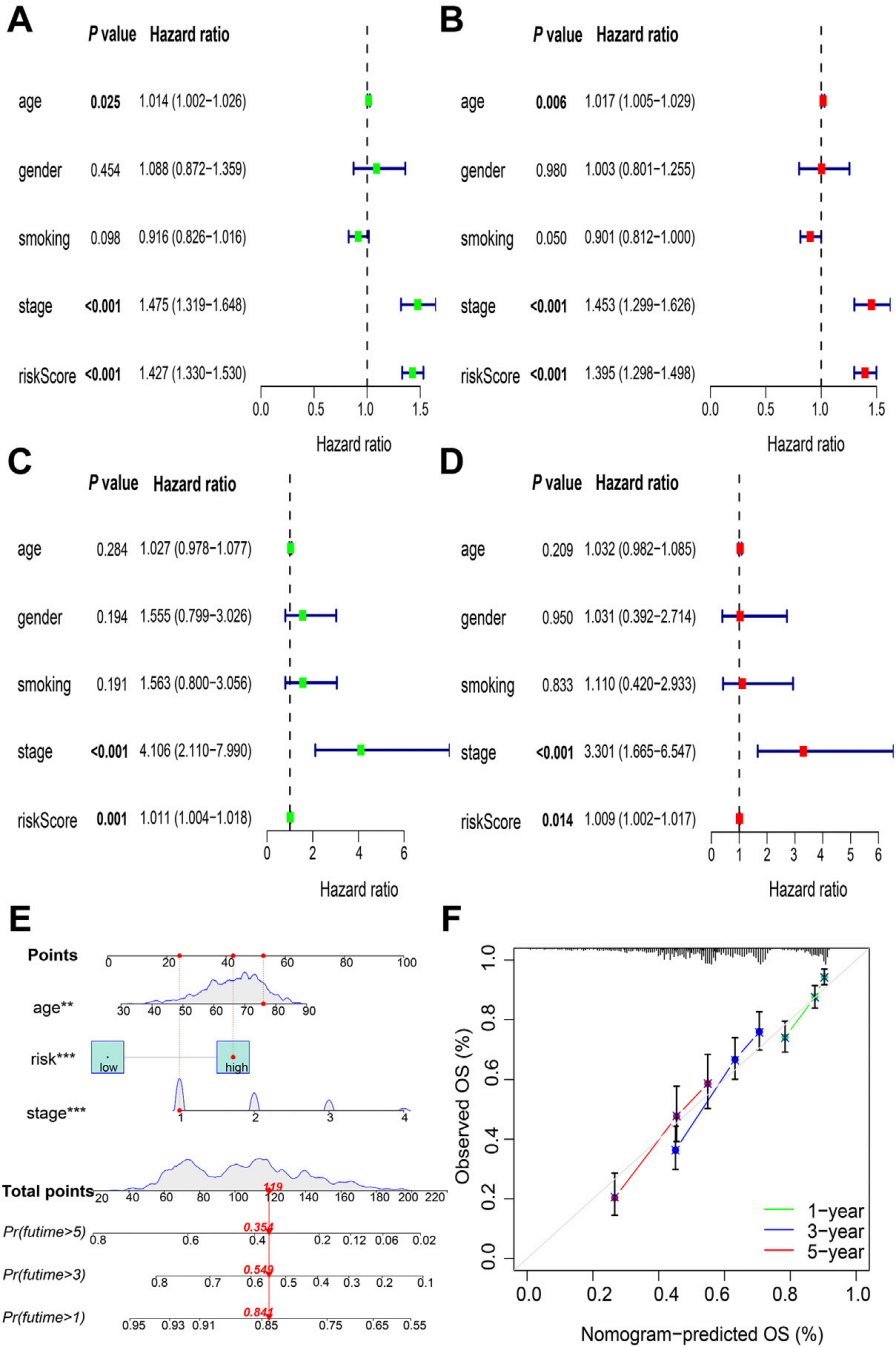
Identification of independent prognostic factors and construction of nomogram. Validation of the risk score as an independent prognostic factor using the univariate Cox analysis in **(A)** TCGA cohorts and **(C)** GSE31210 cohorts, and multivariate Cox analysis in **(B)** TCGA cohorts and **(D)** GSE31210 cohorts **(E)** Nomogram to predict OS at the end of 1-, 3-, and 5-year periods **(F)** Calibration plots of the nomogram. TCGA, The Cancer Genome Atlas; OS, overall survival. **p < 0.01; ***p < 0.001.

Univariate Cox regression analysis showed that the age (hazard ratio [HR] = 1.014; *p* = 0.025), UICC stage (HR = 1.475; *p* < 0.001), and allocated risk score (HR = 1.427; *p* < 0.001) were correlated with prognosis in TCGA cohort (Figure 5A). This was confirmed using multivariate Cox regression analysis (for age: HR = 1.017; *p =* 0.006; for UICC stage: HR = 1.453; *p* < 0.001; for risk score: HR = 1.395; *p* < 0.001) (Figure 5B). Similar results were obtained in GSE31210 when testing was done using univariate Cox regression analysis (for UICC stage: HR = 4.106; *p* < 0.001; for risk score: HR = 1.011; *p* = 0.001) (Figure 5C) and multivariate Cox regression analysis (for UICC stage: HR = 3.301; *p* < 0.001; for risk score: HR = 1.009; *p* = 0.014) (Figure 5D). Thus, risk score can independently predict the prognosis of NSCLC.

### Nomogram prediction model

A nomogram prediction model was generated using independent prognostic factors, including age, UICC stage, and risk score in patients with NSCLC, and the estimated survival probabilities after 1-, 3-, and 5-years were calculated by plotting a vertical line between the total point axis and each prognostic axis (Figure 5E). Calibration plots of the nomogram showed high agreement between the nomogram-predicted and the actual OS at the end of 1-, 3-, and 5-year periods (Figure 5F).

### Results of GSEA

**FIGURE 6:**
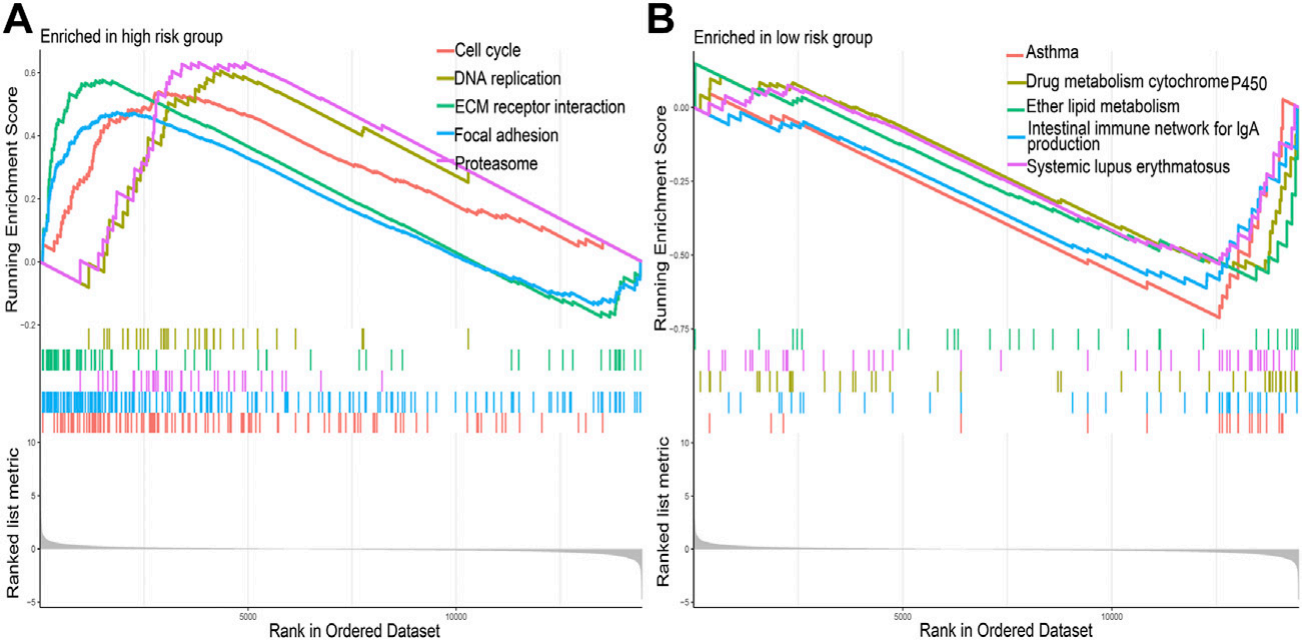
The primary Kyoto Encyclopedia of Genes and Genomes pathways enriched in the **(A)** high-risk group and **(B)** low-risk group.

The results of GSEA showed that DNA replication, cell cycle, and ECM receptor interaction were the main pathways enriched in the high-risk group (Figure 6A); drug metabolism, cytochrome P450, ether lipid metabolism, and intestinal immune network for IgA production were the main pathways enriched in the low-risk group (Figure 6B).

### Results of DNA methylation and mutation

**FIGURE 7:**
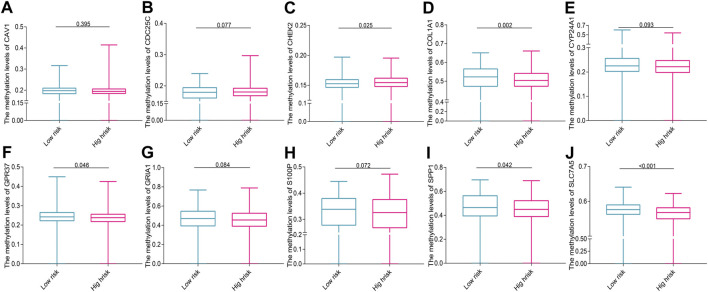
The methylation levels of prognostic genes in the two risk groups **(A)** CAV1, **(B)** CDC25C, **(C)** CHEK2, **(D)** COL1A1, **(E)** CYP24A1, **(F)** GPR37, **(G)** GRIA1, **(H)** S100P, **(I)** SPP1, and **(J)** SLC7A5.

**FIGURE 8:**
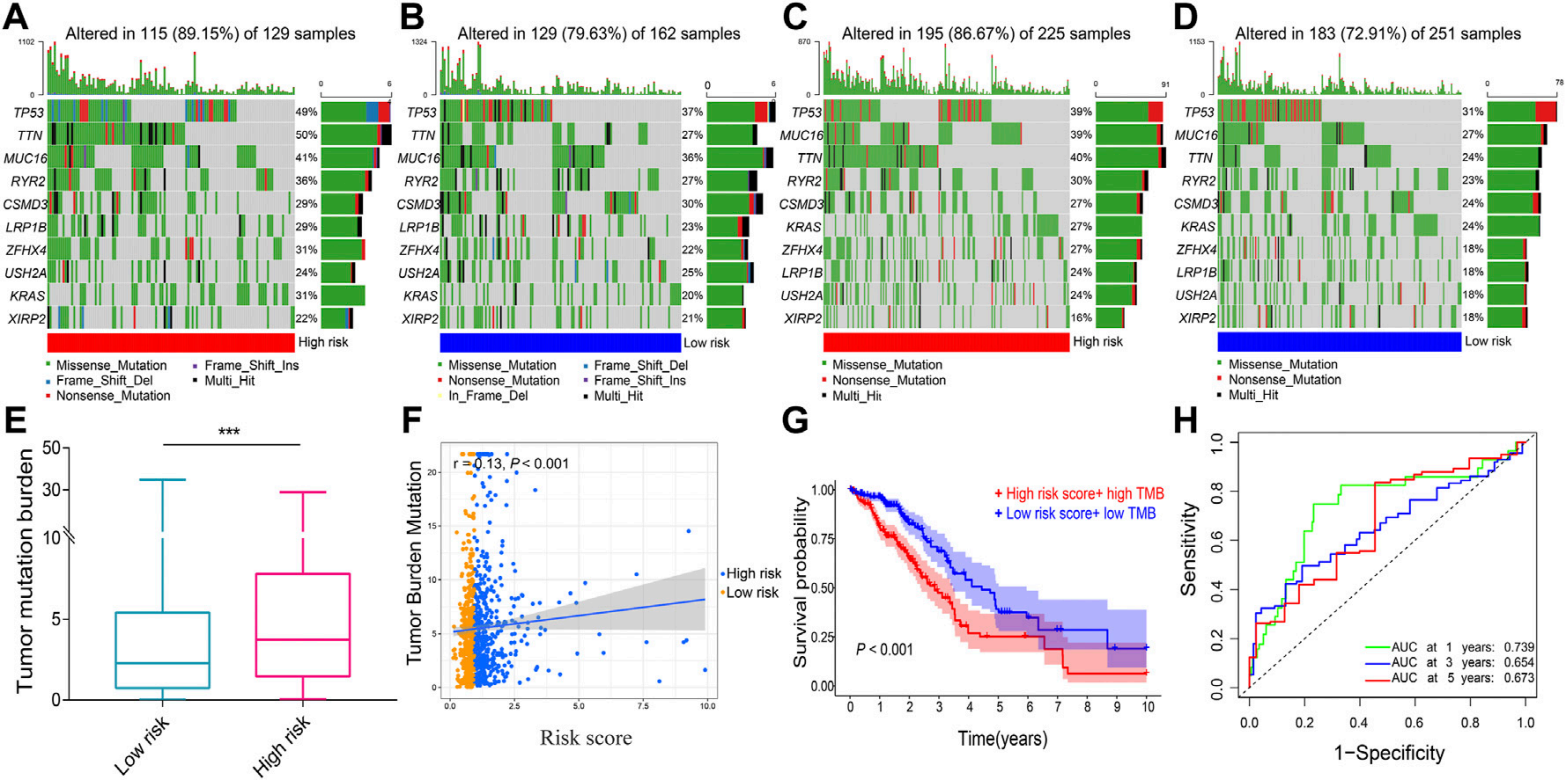
The tumor mutation analysis. Comparison of gene mutation frequencies in the high-risk group of **(A)** LUAD and **(C)** LUSC. Comparison of mutation frequencies in the low-risk group **(B)** LUAD and **(D)** LUSC **(E)** Boxplot illustrated that the TMB value was significantly higher in the high-risk group in NSCLC **(F)** Scatter plot of correlations between the TMB value and the risk score in NSCLC **(G)** Kaplan–Meier survival curve between the high-risk score + high TMB group and the low-risk score + low TMB group **(H)** ROC curve analysis. LUAD, lung adenocarcinoma. LUSC, lung squamous cell carcinoma; NSCLC, Non-small cell lung cancer; TMB, tumor mutation burden; ROC, receiver operating characteristic. AUC, an area under the ROC curve. ***p < 0.001.

A total of 753 overlapping patients with NSCLC were identified between the patients with DNA methylation data and the patients with complete follow-up data, among whom 386 patients were in the low-risk group and 367 patients were in the high-risk group. Methylation data of SCN1A and KLK6 are missing and zero, respectively, and thus are excluded. The overall trend of methylation levels of 10 prognostic genes in the high-risk group was lower than that in the low-risk group (Figures 7A–J). The mutation analysis revealed that TP53, MUC16, TTN, and RYR2 were the most frequently mutated genes in both two risk groups of both LUAD (Figures 8A and B) and LUSC (Figures 8C and D). The high-risk group had a higher TMB compared with the low-risk group in NSCLC (*p* = 0.002, Figure 8E) and a positive correlation existed between risk score and TMB (r = 0.13, *p* < 0.001, Figure 8F). Kaplan–Meier survival curve showed that the patients with the high-risk score and high TMB had a worse OS than those with the low-risk score and low TMB (*p* < 0.001; Figure 8G), and the AUC values for OS at 1-, 3-, 5-year periods were 0.739, 0.654, and 0.673, respectively (Figure 8H).

### Results of immune infiltration

**FIGURE 9:**
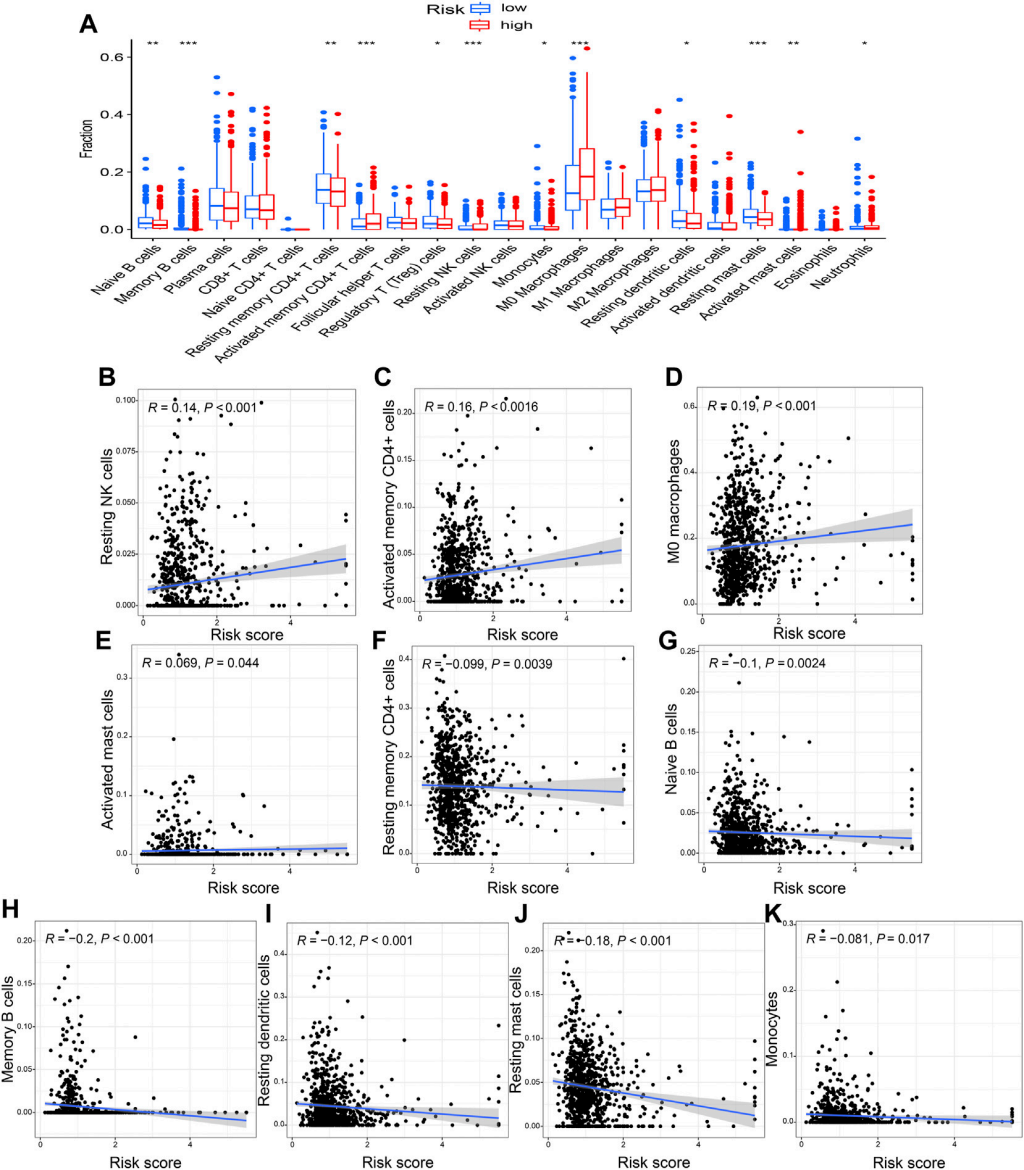
The correlation of risk score with immune infiltration **(A)** The different immune-infiltration profiles between the two risk groups. A positive correlation with the risk score was seen for **(B)** resting NK cells, **(C)** activated memory CD4^+^ T cells, **(D)** M_0_ macrophages, and **(E)** activated mast cells. Whereas **(F)** resting memory CD4^+^ T cells, **(G)** naïve B cells, **(H)** memory B cells, **(I)** resting dendritic cells, **(J)** resting mast cells, and **(K)** monocytes were negatively correlated with the risk score. *p < 0.05; **p < 0.01; ***p < 0.001.

The immune-infiltration profiles between the two risk groups were significantly different and displayed in the violin plot (Figure 9A). The correlation analysis showed that risk score was positively correlated with resting NK cells, activated memory CD4^+^ T cells, M_0_ macrophages, and activated mast cells, and negatively correlated with resting memory CD4^+^ T cells, naive and memory B cells, resting dendritic cells, resting mast cells, and monocytes (*p* < 0.05; Figures 9B–K).

### The risk score correlated with chemotherapy sensitivity

**FIGURE 10:**
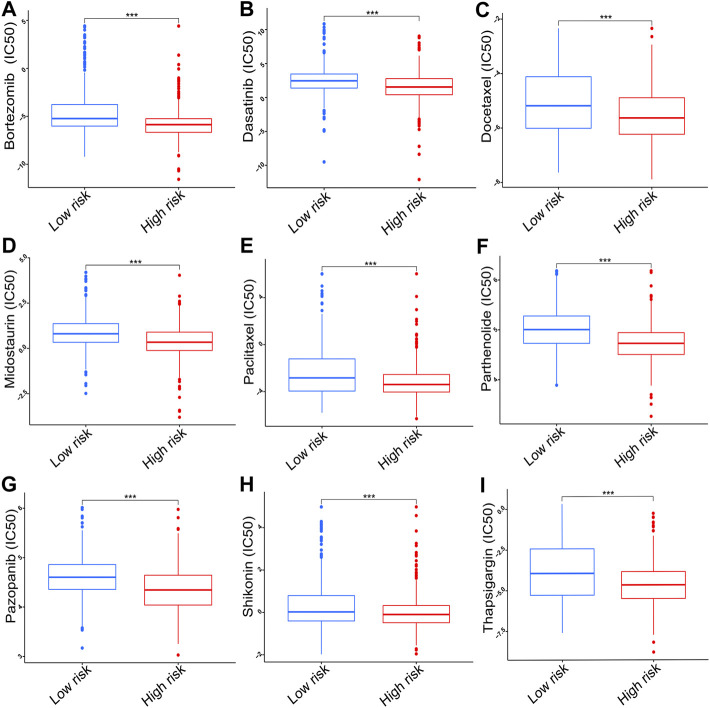
The IC50 of chemotherapy drugs including **(A)** bortezomib, **(B)** dasatinib, **(C)** docetaxel, **(D)** midostaurin, **(E)** paclitaxel, **(F)** parthenolide, **(G)** pazopanib, **(H)** shikonin, and **(I)** thapsigargin. IC50, half inhibitory concentration. ***p < 0.001.

The results of chemotherapy sensitivity analysis indicated that the high-risk group exhibited lower IC50 values for bortezomib, dasatinib, docetaxel, midostaurin, paclitaxel, parthenolide, pazopanib, shikonin, and thapsigargin compared with the low-risk group (*p* < 0.001, Figures 10A–I).

### The Results of real-time PCR

**FIGURE 11:**
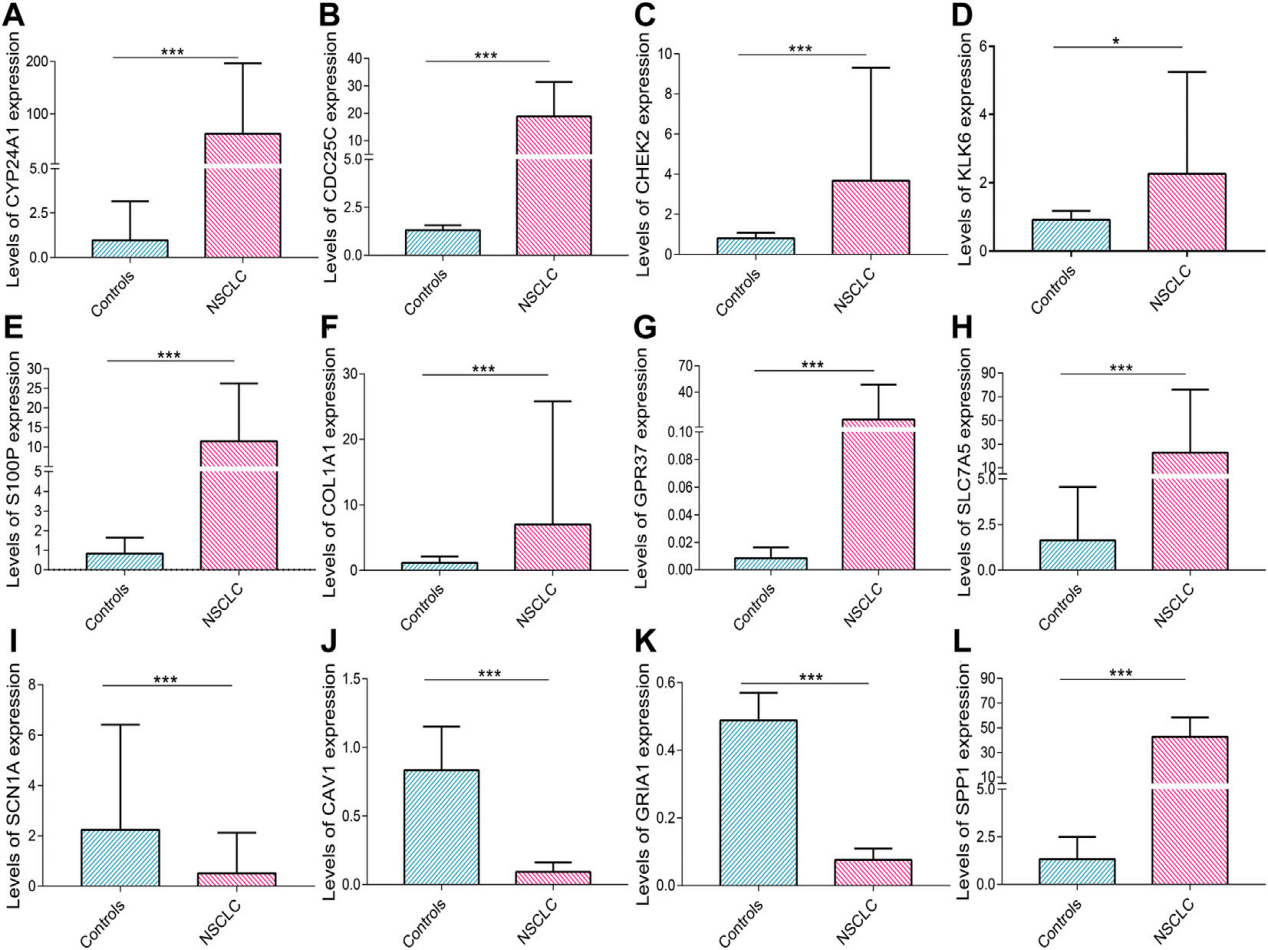
Real-time PCR validation. The levels of **(A)** CYP24A1, **(B)** CDC25C, **(C)** CHEK2, **(D)** KLK6, **(E)** S100P, **(F)** COL1A1, **(G)** GPR37, **(H)** SLC7A5, **(I)** SCN1A, **(J)** CAV1, **(K)** GRIA1, and **(L)** SPP1 mRNA expression in NSCLC. Data are presented as median (interquartile range). NSCLC, non-small cell lung cancer, PCR, polymerase chain reaction. *p < 0.05; **p < 0.01; ***p < 0.001.

The levels of CYP24A1 (*p* < 0.001), CDC25C (*p* < 0.001), CHEK2 (*p* < 0.001), KLK6 (*p* = 0.044), S100P (*p* < 0.001), COL1A1 (*p* < 0.001), GPR37 (*p* < 0.001), and SLC7A5 (*p* < 0.001) mRNA expression were higher in cancerous tissues of patients with NSCLC than in corresponding controls (Figures 11A–H); whereas the levels of SCN1A (*p* < 0.001), CAV1 (*p* < 0.001), and GRIA1 (*p* < 0.001) mRNA expression in cancerous tissues of patients with NSCLC were lower than those in control tissues (Figures 11I–K). The increased SPP1 mRNA levels in NSCLC were validated in previous research (Miao et al., 2021) (Figure 11L).

## Discussion

In the present study, a prognostic signature was successfully established using genes involved in redox homeostasis. Kaplan–Meier survival curve and ROC curve analysis validated the predictive accuracy of the signature. Furthermore, the constructed nomogram accurately predicted the OS of patients, and there was high conformity between the predicted and actual OS. The risk score is an independent prognostic factor for NSCLC and is correlated with tumor progression, immune infiltration, DNA methylation, TMB, and chemotherapy sensitivity.

The mechanism(s) of action of genes associated with redox homeostasis in the progression and prognosis of NSCLC remains unclear. CDC25C mRNA expression is increased in LUAD and associated with poor prognosis (Xia et al., 2019). KLK6 mRNA expression is enhanced in patients with lung cancer (Ju et al., 2020) and is an indicator of tumor proliferation and poor OS (Nathalie et al., 2009; Michel et al., 2014). The overexpression of the SPP1 was associated with the growth and stage of lung cancer, and lymph node metastasis (Shijubo et al., 1999; Schneider et al., 2004; Boldrini et al., 2005; Donati et al., 2005; Hu et al., 2005). CAV1 is downregulated in lung cancer and thus acts as a tumor suppressor gene (Bélanger et al., 2004; Zhan et al., 2012). CYP24A1 overexpression is associated with accelerated cell growth and invasion (Shiratsuchi et al., 2017) and worse survival in LUAD (Chen et al., 2011), and its polymorphisms are associated with susceptibility to lung cancer (Qu et al., 2019; Xiong et al., 2020). The levels of S100P mRNA and protein expression are higher in lung cancer, and upregulated S100P increases cancer cell migration and metastasis (Hsu et al., 2015). The levels of SLC7A5 mRNA and protein expression are increased in NSCLC and are correlated with grade, pathologic stage, and OS (Takeuchi et al., 2010; Liu et al., 2021). The levels of COL1A1 mRNA expression in lung cancer are increased and are significantly correlated with tumor diameter, metastasis, and OS (Geng et al., 2021; Hou et al., 2021). Our real-time PCR validated dysregulated CDC25C, KLK6, SPP1, CAV1, CYP24A1, S100P, SLC7A5, and COL1A1 expression in NSCLC. Additionally, GPR37 expression is lower in cancer and is a prognostic indicator (Liu et al., 2014). Elevated GRIA1 mRNA levels are significantly associated with worse OS in patients with basal-like urothelial carcinomas (Tilley et al., 2017). CHEK2 is identified as a susceptibility gene for pheochromocytomas and paragangliomas (Gao et al., 2021), and CHEK2 mutations are associated with the survival of patients with stage T1 bladder cancer (Złowocka-Perłowska et al., 2021). SCN1A has not been reported to be involved in cancer origin and progression. The upregulated levels of GPR37 and CHEK2 expressions and reduced levels of GRIA1 and SCN1A expressions in NSCLC were firstly reported in this study. However, the exact molecular mechanisms remain unknown, and further investigations are needed to elucidate the possible mechanisms.

Previous studies have reported the successful construction of the redox-related prognostic signature in clear cell renal cell carcinoma (Wu et al., 2021b), and hepatocellular carcinoma (Tu et al., 2021). However, both of them are only based on bioinformatics analysis and thus, lack reliable experimental verification. Additionally, a redox-related gene signature has been established in LUAD (Xiao et al., 2022b), which is only suitable for a subset of patients with NSCLC and also lacks reliable experimental verification. In the present study, the redox-related gene signature was first constructed in all the patients with NSCLC, and Kaplan–Meier survival curve showed that the high-risk group had a worse OS than the low-risk group, which was different from and cannot be replaced by the previously constructed signature using hypoxia-related gene (Chen et al., 2022), pyroptosis-related gene (Zhang and Yan, 2021; Li et al., 2022), autophagy-related gene (Wu et al., 2021a), and inflammatory response-related gene (Zou et al., 2021) because it could be meaningful in exploring the value of the redox-related gene in initiation, progression, prognosis, and therapy of NSCLC. The previous prognostic signatures in other cancers showed better diagnostic performance, with an AUC value of more than 0.8 (Qian et al., 2022; Zhang et al., 2022). However, in the current study, the AUC value for OS at the 5 year periods was 0.657 in TCGA and 0.717 in GSE31210. Various researches showed that the AUC values of many signatures for lung cancer or NSCLC were more than 0.75 (Wu et al., 2021a; Li et al., 2021b; Ouyang et al., 2021; Qu et al., 2021; Zhang and Yan, 2021; Zou et al., 2021; Wang et al., 2022b; Chen et al., 2022; Li et al., 2022; Yang et al., 2022). Thus, the prognostic biomarkers or signatures with a higher AUC value need to be explored in the future. Moreover, the 12 genes in prognostic signature were validated using real-time PCR in NSCLC; thus its reliability is higher than the above-mentioned redox-related gene signature for clear cell renal cell carcinoma (Wu et al., 2021b), hepatocellular carcinoma (Tu et al., 2021), LUAD (Xiao et al., 2022b), and hypoxia-related gene signature (Chen et al., 2022), pyroptosis-related gene signature (Zhang and Yan, 2021; Li et al., 2022), autophagy-related gene signature (Wu et al., 2021a), and inflammatory response-related signature (Zou et al., 2021) for NSCLC. Moreover, the risk score positively correlated with the progression of NSCLC and was an independent prognostic factor for NSCLC adjusted for age and UICC stage. Importantly, the signature was successfully validated using multiple GEO datasets. Therefore, our prognostic signature can successfully predict prognosis and is not restricted by different groups of patients, sequence platforms, and technologies. These were consistent with the successfully established signatures for NSCLC (Ye et al., 2021; Yang et al., 2022). Finally, a nomogram prediction model was constructed using the risk score, age, and UICC stage to precisely predict OS rate after 1-, 3-, and 5-year periods, and calibration plots of the nomogram showed high agreement between the nomogram-predicted and the actual OS.

The mechanisms of different prognoses of two risk groups were explored using GSEA, and findings showed that DNA replication and cell cycle were the main pathways in the high-risk group. The cell cycle has a vital role in lung cancer pathogenesis and progression (Caputi et al., 2005), and DNA damage-response or DNA-repair genes with germline aberrations induce cancerous tendencies (Brown et al., 2017). Thus, the worse OS in the high-risk group may be correlated with DNA replication and cell cycle. Additionally, DNA methylation alteration could result in cancer development and progression (Kinnaird et al., 2016). Hypomethylation of the promoter is correlated with the overexpression of oncogenes and other genes associated with tumor invasion or metastasis (Ehrlich, 2009). Our results showed that methylation levels of 10 prognostic genes were lower in the high-risk group. Moreover, dysregulated TMB has also been reported to be involved in the prognosis of cancers (Handy and Loscalzo, 2012), and could predict immunotherapy response (Chalmers et al., 2017). The high TMB is correlated with poor prognosis in LUAD (Zhao et al., 2021b). The high-risk group has an increased TMB compared with the low-risk group in the present study, and Kaplan–Meier survival curve showed that the patients with the high-risk score and high TMB had a worse OS than those with the low-risk score and low TMB. Thus, DNA methylation and mutation play a critical role in the progression and prognosis of NSCLC.

In recent years, immunotherapy has become an important therapeutic method for multiple tumor types (Alemohammad et al., 2021; Cai et al., 2021); thus, a possible correlation between the risk score and immune infiltration was assessed. The results showed that a high-risk score was positively correlated with macrophages, activated memory CD4^+^ T cells, activated mast cells, and resting NK cells. Tumor-associated macrophages (TAMs) are significantly correlated with angiogenesis, progression (Hwang et al., 2020), and a poor prognosis (Montuenga and Pio, 2007; Li et al., 2020b) in NSCLC. A high percentage of CD4^+^ T cells in the tumor stroma is associated with a poor prognosis in lung cancer (Giatromanolaki et al., 2021). The mast cells were activated in lung cancer by cancer-derived extracellular vesicles (Salamon et al., 2020), and activated mast cells accelerated cancer cell proliferation and migration (Xiao et al., 2014; Xiao et al., 2019). Thus, our results indicated that the high-risk group has a worse OS via increased immune infiltration.

Considering that chemotherapy is still an important therapeutic method for advanced lung cancer, a possible association between the risk score and chemotherapy was also studied. The results showed that the high-risk group had a lower IC50 value for nine chemotherapy drugs, suggesting that the high-risk group may respond better to chemotherapy. Thus, our prognostic signature can be useful for guiding treatment strategies for patients diagnosed with NSCLC.

However, it is worth noting that the results obtained in this study were based on bioinformatic analyses of the public database, which needs to be validated in a clinical prospective, multicenter study. Furthermore, studies are required to validate molecular mechanisms between two risk groups in NSCLC. Moreover, the previous studies showed that the prognostic signature could be used to guide immunotherapy choices (Cao et al., 2022). However, there was not a significant correlation between the risk scores and levels of immune checkpoint inhibitors expression. Despite these limitations, our study has established a promising signature for predicting patient prognosis. The signature was validated using GSE31210, GSE68465, and GSE26939 datasets. Secondly, the 12 prognostic genes were validated using real-time PCR in lung tissue samples. Lastly, the risk score was shown to be correlated with immune infiltration and chemotherapy response; thus, it could be applied to guide treatment modules in the clinical setting.

## Conclusion

A redox-related gene signature was established using LASSO regression analysis and was represented as a risk score. The high-risk score in this signature was significantly correlated with worse OS and the risk score was an independent prognostic factor for NSCLC. Moreover, the risk score was correlated with tumor progression, DNA methylation, TMB, immune infiltration, and sensitivity to chemotherapy.

## Data Availability

The original contributions presented in the study are included in the article/Supplementary Material; further inquiries can be directed to the corresponding author.
