# Supplementary material for: Construction of a redox-related gene signature for overall survival prediction and immune infiltration in non-small-cell lung cancer
Source: Front Mol Biosci. 2022 Aug 16;9:942402. doi: 10.3389/fmolb.2022.942402 (PMC9425056; doi:10.3389/fmolb.2022.942402)
Supplement: Supplementary file 1 [file Table1.DOCX]

TXN

APEX1

PC

GSR

GLRX2

TXNRD2

TXNRD1

NMRAL1

GLRX

P4HB

GPX1

PRDX5

NFE2L2

CISD1

PRDX6

HMGB1

SELENOT

TXNRD3

PRDX1

TXNL1

PRXL2A

NXN

PRDX2

SELENON

SOD1

TXN2

HIF1A

SELENOO

TXNIP

PARK7

PRDX4

CAT

NOX5

PRDX3

NOX4

SELENOH

SELENOW

JUN

KEAP1

PDILT

CBS

SOD2

NQO1

ERO1B

GLRX3

RNF7

ERO1A

CHCHD4

HMGB3

TMX1

CYCS

GPX4

TMX3

SELENOV

ARNTL

PDIA2

HMGB2

NOS3

SCO2

MPO

UQCRFS1

HMOX1

CLOCK

NPAS2

NMRAL2P

TNF

FDX1

TP53

EPAS1

G6PD

XDH

GFER

MAPK8

NOS1

MICAL1

CASP3

PTPN1

ACP1

CYBB

ALDH5A1

TXNDC17

MAPK14

ATF4

PDIA3

PIR

POR

GLO1

TXNDC11

TMX2

NOS2

DNAJC10

CLIC4

CISD2

MPST

SENP3

GPX7

SLC8B1

TXNDC2

TXNDC8

CLIC1

MAPK1

DLD

CLIC2

NGB

SELENOF

GCLC

MIEN1

NDOR1

LGALS9

UQCRC2

RNH1

HRAS

UQCRC1

MACROH2A1

COX19

GPX3

SLC7A11

SLC52A1

TPI1

CYBA

DHODH

RAC1

VKORC1

CDKN3

HAO1

MAP3K5

COQ2

ERP44

RYR1

CXCL8

CYC1

FOXO1

NFE2L1

NCF1

NOX1

NR1D2

MB

GAPDH

ATP5IF1

LPO

APP

GOT2

TXNDC5

PTS

CYB5R4

PITRM1

UQCR10

FOS

ITPR1

GPX2

KRIT1

LIPT2

H6PD

UQCRB

ATG4A

UQCRQ

ATG4B

UQCRH

UQCR11

AQP11

DNAJC24

CYB561D2

PDIA6

EGF

VEGFA

RYR2

CCL2

SETD2

BCL2

HTATIP2

RNF41

LACC1

CBSL

CYB5A

GGT1

FAS

DDIT3

QSOX1

ICAM1

NNT

PDIA4

ALB

NDUFA2

MT-CYB

GSTP1

HSCB

AKR1B1

IL1B

NCF2

NAPRT

PTEN

SOD3

MIF

NFKB1

OMA1

HBG2

STAT3

SMPD3

TGFB1

CP

NADSYN1

MAPK10

SNCA

PARP1

SHC1

NUP62

MTR

PRNP

FKBP1B

EGFR

HSPA4

SELENOS

CDKN1A

CDC25C

SIRT2

AKT1

BAX

RELA

GSS

VCAM1

MSRB1

NFKBIA

AIFM1

SRC

ARHGDIA

HVCN1

PRKAA1

ATOX1

MMP9

ARNT

CASP9

CHUK

ATM

MTOR

CYP17A1

TF

ALOX5

EGLN2

AGT

HMOX2

ESR1

NDUFS4

MMP1

EDN1

PTPN3

DUSP19

TAPBP

HSF1

INTS2

SP1

INTS3

AKR1A1

TYR

CD44

PTP4A1

BCL2L1

HSPA8

PAX8

CREB1

PTGS2

HSPA5

GLUD1

BCAT2

ACO1

GPX8

SMPD1

PTK2

SDHA

EGR1

FMO2

APOE

RHOA

VKORC1L1

ORAI1

SIRT1

LGALS1

KIR2DS4

PDGFRB

MAOA

XRCC5

BACH1

QSOX2

ATP2A2

BIRC5

NUP155

AHR

CTSB

IFNG

CD4

PIK3CG

IL4

COX5A

ALDH9A1

AQP9

FMO5

CTBP1

MT3

ADH5

SUMF1

ERP27

CANX

OLR1

PTK2B

GSTM1

PKM

GABPA

NR3C1

ETFDH

SGCB

PDIA5

TFRC

VDAC1

IREB2

CALR

GCLM

INSR

SORD

NME8

LCK

ABCC1

CYB5R3

SIRT3

RPA1

TRPM2

BLVRB

OXA1L

DNM1L

NCF4

TXNDC12

NXNL1

TMX4

SQSTM1

RANBP2

CSNK2A1

ODC1

RPS27A

ORAI3

SLC2A10

PTGES2

NME9

CASP8

CD40

ENO1

ATP7A

IDO1

FOXO3

SRXN1

INS

OGG1

MMP2

HSPB1

GRB2

IL1A

CDC42

ENOX2

PPARG

SLC11A1

ARHGDIB

GLRX5

TXNDC9

SH3BGRL3

TXNDC16

BRCA1

FXN

CRAT

PDR

CCND1

MAPK7

PYCR1

MAOB

DYNLL1

MAPK3

KCNH2

CTSK

RORA

GRIA1

CPT2

IL2RG

RPS19

TUBA1A

GHSR

GJB2

GLDC

MC1R

BMP2

MITF

DLG4

DDX3X

CSNK2A2

IFIH1

PIK3C2A

PROS1

MSX2

YAP1

F9

ALPP

FST

CLTC

MSR1

SLC25A20

SIGMAR1

PTGER3

UNG

TCF4

GPD2

WNT1

C1QBP

CCR1

AQP4

FDPS

ANGPTL4

BCL2L11

BMP1

BCKDK

MDH1

ABCD1

ACACB

HPD

GSTM3

HNRNPA2B1

HNRNPK

EEF1A2

NEDD4

PPP2R2B

IDH3B

IGFBP7

OPRK1

IL6ST

RAN

KPNA2

KLF4

KRT19

TWIST1

XPO1

ALOX5AP

FABP4

BAK1

FADS1

LTB4R

ALDH4A1

MCM7

FUS

CPT1B

HNF1B

DGUOK

RPL15

NDUFS2

NONO

SDHC

NLRP1

RPS10

NTF4

TPM2

KMT2A

KITLG

PTRH2

SF3B1

PBRM1

THBD

LIAS

VNN1

WARS2

VTN

ARPC3

ANXA6

ARHGEF7

BCKDHB

ADH1B

FMO1

CDX2

COQ7

DLG1

MYO1E

RPL18

RPL21

NAT2

DBT

NDUFA9

SFPQ

SIRPA

SELENBP1

IFNGR2

IGFBP1

IGFBP4

SNW1

NUP214

SLC39A14

SLC39A4

PTPN7

ITM2B

TIRAP

VAPA

XRN2

CCS

EXOSC3

ANKRD1

ACTC1

ACTR3

COQ6

DMGDH

NCL

EIF4G2

HNRNPU

PPP1R12A

PREP

SAA1

RPS7

SARDH

NFIX

RPL7A

PNKD

SLC31A1

NUP98

NTSR1

LASP1

TRPC1

TCOF1

PYY

KRT10

GCG

MAP4

F2RL2

FBXO7

FANCE

BCL3

FLII

FOSL2

ERCC8

HNRNPC

MLXIPL

DUSP2

DUSP5

MYL6

MPV17

RNF13

GRP

CSRP1

CRY2

PAPPA

PASK

PCBP1

SNRPE

RPS15

RPS3A

RPS5

PTGES

RAB35

KHSRP

TRPV2

RPL23A

PPA2

TFF3

KRT7

ABLIM1

APLP1

AGXT2

FKBP3

CRYGD

CTDSPL

CAPZA1

COTL1

CKAP5

CHDH

DIO3

ECH1

CD276

IGF2BP1

NME6

RPLP1

RPS16

RPS18

NTS

TIMP4

STEAP1

STK25

SNRPD2

KRT2

LMO7

BDH2

MIOX

FIS1

AKAP1

AKAP10

ACMSD

GAN

GUCA2B

COX6C

HNRNPM

HNRNPR

DHRS2

RPL18A

CDC5L

EHD2

RPL24

CRELD1

SIPA1L3

SF3A1

RPL6

RPS11

SLC25A37

SRSF7

SRSF9

RBP1

MT1F

ZNF638

VIL1

YBX3

UBD

ZNF143

CBR4

LYRM4

LRRFIP2

BMS1

MRPS12

MYL12B

DEFA5

DDX24

RNLS

NMNAT3

SLC31A2

SRP14

ILF3

SSBP3

SYNCRIP

TDRKH

SF3B2

EML4

EXOSC4

CEBPZ

HNRNPA3

COX7A1

DSPP

GRK2

DDX27

GRIN3A

SIPA1L1

NUP35

NUP205

LGALS7

STAU1

MT1H

RPL17

DOCK5

SF3A3

RACK1

IMP3

PGP

RCL1

G3BP2

FTSJ3

COQ8A

DHRS11

DUOXA1

NOP2

NPM3

PRODH2

LAMTOR5

TMBIM6

RBM14

ARPC5L

MCUR1

RRP15

SLC25A30

OSGIN1

VARS1

ARSH

ACTBL2

PPTC7

RRS1

TMEM33

PDCD11

NOL7

ATP5F1B

AHNAK2

FOXRED2

H1-4

PRRC2C

SLTM

SRRM2

LARP4

RBM34

COQ8B

MT-ND6

VOPP1

MCMBP

RMDN1

CGB3

H4C1

SELENOI

PATJ

TMEM241

MROH7

ATP5MC3

H1-3

MT-CO3

SEPTIN4

CYRIB

CARD19

TERC

H4C14

SEPTIN11

H2BC13

MIR200A

NCF1C

MIR433

MIR205

RN7SK

MIR382

LOC110467515

LOC107548112

ERBB4

FGFR2

EZH2

BRAF

MET

CHEK2

FLT4

CDK5

TLR3

ATR

ADA

GRIN2A

PCSK9

PRKCG

PRKACA

VDR

GNAS

ESRRB

CCND2

DRD2

CDH1

GRIN2B

CDH2

PRKCH

PFKM

NR5A1

NGF

SMARCA4

RARB

TBXAS1

TEK

STAT6

ROCK1

VLDLR

SLC25A13

STAR

KLK3

STK3

ALDH18A1

ACLY

CKB

HADHA

DFFA

EHMT2

PDHB

NR1I2

NPHS1

IDH3A

PPM1A

HSD17B10

ISL1

TOP2B

STUB1

TDO2

DLST

ADM

ATP6AP2

AHSG

ABCD3

BRD4

CNDP1

DYNC1H1

NDUFV2

IL12B

PFKFB3

SDHD

RPL35A

OGT

SLC17A5

ITPKB

PYCR2

IL3

NDUFA10

PLAUR

USP1

GDI1

GNE

ANP32A

AGTR2

ACAA1

GSTO1

MMACHC

NDUFS6

EIF5A

HTRA1

PAX4

SLC37A4

SLC25A11

PSME2

TSFM

TAGLN

IYD

TFAM

LRPPRC

AARS2

COX15

EEF1D

RPN1

SNCG

TRIM21

CUL2

TFEB

CCK

ADNP

BMP15

FLOT2

BHMT

BRD3

CHKA

MRPS22

MRE11

EIF5

COPA

PPIC

S100A11

NFKBIB

OSGEP

SND1

PTPRT

UBE2M

PTP4A2

TRMT1

XRCC3

GNL3

CCT7

EPS15L1

FKBP2

MBD2

CYBRD1

GSTK1

DNAJB11

ECI2

IL19

PCMT1

SART1

HSPB2

UBE2O

SUMO2

PECAM1

PARP3

JMJD1C

PSMC4

CD53

CKMT1B

CHCHD2

MRPL13

MYBBP1A

DAP3

NES

SFXN3

DHRS9

MEMO1

ABCE1

DNAJC7

MRPS17

MRPS9

NACA

KARS1

GOLGB1

ARL6IP5

ABHD10

BTG3

CKMT1A

MRPS35

SP140

PLP2

TAF5

DUSP12

SUB1

ZC4H2

ATAD3B

AHNAK

MCU

BRF2

GRPEL2

MRPL14

MRPL2

MRPL23

DERL1

MRPL49

MRPS11

MRPS21

MRPS31

PFDN2

HSD17B13

TOMM22

TRMT10C

HSPA13

YARS1

YEATS2

ZBTB33

MARS1

MGME1

CXorf56

SARS1

TOX4

RARS1

TBPL2

PPARGC1B

SCML2

HSDL2

PGAM5

MRPL50

ZNF280C

ERVW-1

EMC2

ATP5PD

CISD3

ATP5MF

GATD3A

IGHE

GAS5

FGFR1

BLK

RAD50

YWHAG

F10

ATP2A1

BLM

GALK1

HPRT1

PDHA1

EGLN1

ENO3

PIP5K1C

TOP1

STXBP1

VCP

YWHAB

MAT1A

MAT2A

APRT

FGA

BUB3

MALT1

LYZ

HEXB

MTAP

GRN

CSNK2B

PPP2R1A

IMPDH2

UBE2N

YWHAH

ARF1

ASNS

FEN1

FOLH1

CES1

DRD4

DKC1

PRKAR2A

PAX5

UBA1

SUMO1

PSMA6

PRPS1

GNB1

F2RL1

ABCB11

CPS1

HINT1

DSG2

MYH11

SAE1

SLC5A5

SMARCB1

SMARCE1

SMC3

PMVK

UBE2D1

SRPK1

PCSK1

EIF2AK2

MYO6

TGIF1

UROD

USP14

WRN

CASP14

CALM2

APOH

ATP6V1A

ATP6V1B1

FMR1

CTPS1

GANAB

AP1B1

HMGA2

CHD4

MTM1

DGKG

RPA2

EIF2B2

NCOA1

NCOA2

PSMA4

PEBP1

PPM1B

SOAT1

RUVBL1

RUVBL2

NHP2

NSF

TAP2

TMED10

HUWE1

PRPH

MAP4K1

AP1S2

ETF1

ATP6V0D1

FBL

AIMP1

ACTR2

FOXO4

FSCN1

CYP4A11

CLCN4

HAX1

DSG1

CD82

DDX1

EIF2B1

EIF4A3

RPL26

CORO1A

PRKCSH

IL21

NOP56

SEC23A

POU3F2

PPM1G

PDE3B

POLR2E

PLP1

SLC25A6

OLA1

PTGES3

PSMD7

UBA2

PTPN13

RBBP4

RANBP1

RCAN1

KIF1A

TECR

RAB10

PSMD2

POLR1C

HSPH1

LMNB2

CALM3

ANXA7

MBD4

ATXN10

CNP

MTA1

DTYMK

CALD1

EIF3F

NEFM

PSMA2

PRPF4

PAICS

PALLD

PDCD6IP

SMN2

SENP1

NSUN2

PSMC1

SSB

UCK2

UBE2V1

NAA10

TAB1

VTI1B

CAP1

GART

ATG3

ARCN1

FAM20C

MAFA

ALDOC

AIMP2

ERLIN1

FLOT1

ACOT7

BRD7

LYVE1

CLTB

CHMP4B

MYL1

CDC37

GPLD1

EIF3A

EHD1

EIF1AX

PFN2

PRPF6

NPHS2

PABPC4

PAFAH1B3

SNX1

SAR1A

RPN2

USP47

ZFP36

GNLY

CCDC90B

ACAD10

ACSF2

FASTKD2

CST7

HINT2

COX7C

HEY2

CLSTN1

CIRBP

HMGN2

MTPAP

MRPL15

MRPS2

MRPS28

MUL1

DUSP23

MYL7

PDP2

PDPR

NRBF2

SH3PXD2B

SLC7A3

SLC25A26

SLC25A27

SLC25A28

POFUT2

SLC30A6

SLC25A42

SLC25A25

SHPK

NPRL3

IQCE

ISCA1

TOMM34

LGALS4

TET1

SURF4

TMEM70

PHYKPL

DRG2

MYO5C

NADK2

HAO2

HIGD1A

MRS2

TIMM44

CAND1

GATC

GHITM

CCL26

AURKAIP1

ATPAF1

ATPAF2

ARPP19

EXOG

ARMC4

FAHD1

FAM136A

LYRM7

ADO

ADPRH

AIFM3

METAP1D

ACOT13

ACOT2

ADHFE1

ACSM2A

ADCK1

BTBD10

C1GALT1C1

RMND1

CLGN

HEPHL1

GLOD4

GUF1

COX14

COPS6

COMTD1

CHAC1

CHD6

DNAJB4

DNAJC15

DNAJC27

MTHFD2L

MRPL17

MRPL19

MRPL24

MRPL28

MRPL40

MRPL42

MRPL46

MRPS23

MRPS25

MRPS34

DHRS1

MRPS6

DPM2

MTCH1

DUSP15

ECHDC1

DEFA6

DEFB4A

MRM1

NDFIP1

NDUFA7

IFNA5

HTRA3

NMRK1

NIT1

PLA2G15

SLC25A33

OMP

NUDT6

SLC25A16

SLC25A23

NUDT15

SLC15A3

ILVBL

PTGR2

PHLDA2

PHPT1

TPPP3

UGGT1

UGGT2

TSHZ3

STARD7

RAB32

KIAA1549

TCF20

RDH14

SUPV3L1

SUN2

SH3BGRL

SAMM50

PACRG

PCDH1

REXO2

TFB2M

TIMM8B

TIMM9

ZDHHC6

ZNF3

VPREB1

ZADH2

CARD16

GATB

GARS1

MICU2

ANO9

METTL7B

BLOC1S1

ABCA9

ABHD11

ADCK5

ACAD11

AVEN

RMND5A

APOC4

GRSF1

RILP

COQ10B

DPY19L3

MTRF1

MTFR1

MRPL16

MRPL18

MRPL20

MRPL32

MRPL39

MRPL43

MRPS10

MRPS18A

MRPS18C

MRPS24

MRPS27

MRPS30

MRPS33

DHRS7

DUSP18

DUSP26

DUSP8

MYL10

EEFSEC

EFHD1

MOBP

DCAKD

EI24

NDUFB5

IFNA6

PAK5

SNPH

PRELID1

PRICKLE4

SCARA3

SCCPDH

RPUSD4

RSAD1

NFXL1

NIPSNAP3A

OSGEPL1

SLC25A35

SLC25A40

NUTF2

SLC39A2

NT5M

PHYHIPL

TONSL

TMTC1

TMEM14C

TOMM7

LACTB

LACTB2

LANCL1

LETMD1

LECT2

PYROXD1

SUGCT

TMEM230

DUS2

MRPL10

DHX29

KCNK17

JAGN1

RAB24

THEM5

TIMM22

TIMMDC1

ZNF346

CABP2

CCL15

CCDC136

FAM162A

FITM2

ARMC1

ARMC10

LRRC8D

ADCK2

CPQ

HEMK1

CNN3

COX18

CMC1

HMBOX1

CPEB2

CHERP

MMUT

MTRF1L

DNAJC11

MTG1

MTG2

MRPL21

MRPL22

MRPL27

MRPL30

MRPL35

MRPL36

MRPL37

MRPL4

MRPL48

MRPS15

MTIF3

MRPS26

MXRA8

GLYATL1

DEDD

DDX28

MPV17L2

NDUFAF6

IFNA21

IBA57

OXNAD1

IFNA13

PDE12

NIPSNAP3B

SLC48A1

SLC25A44

SLC25A47

OCIAD1

SPRYD4

PIGY

PTCD3

TRIAP1

UQCC2

TBRG4

TATDN3

JCHAIN

TMEM176A

TMEM11

TP53I11

DUSP11

CHCHD7

NPAT

DACT2

TIMM10B

ZNF622

YIF1A

LYRM1

CTU1

GTPBP8

RHBDD3

COA3

MTERF1

MRPL34

MRPL45

MRPL51

MRPL9

ECHDC3

NAXE

NDUFAF7

GRINA

IFNA4

IFNA8

IFNA10

NOA1

SLC25A2

NUDT19

SPTSSA

PTCD2

INTS12

TRIM56

JPH4

TMEM120A

NARS1

STING1

YBEY

ADM2

GET3

CCDC12

B3GLCT

ASIC5

ACSM4

C15orf48

CYB561A3

RMDN3

GUCY1B1

COP1

CHAC2

CHCHD1

HDDC2

MTERF3

DNAJC30

MRPL47

MRPL54

MRPL55

MT4

DCAF5

PRSS35

SERHL2

IFNA7

HSDL1

SLC25A51

NUDT8

NUDT13

TOMM40L

TWNK

UQCC1

PTRH1

TMBIM4

TMEM65

TMEM45B

TMEM186

IFNA17

NAF1

HIGD2A

CACUL1

MIEF1

METTL17

FSIP2

BOLA2

BOLA2B

H2AC4

PDGFB

RPS6KB1

CYP11B1

SLC25A4

RRM1

SLC11A2

RAB11A

TKT

CRYAB

CFH

AOC3

ARF6

GADD45A

MUTYH

IL13

BMP6

BCAR1

LIF

DDAH1

APOA4

HBA1

CDIPT

IMMT

DAP

FBXL5

GPX6

ZBED1

MT-CO2

IGF1R

MAP2K2

AR

FLT1

PRKG1

HSD11B1

MYH9

DUSP6

REN

ATF6

ATP7B

EPHX2

DIABLO

SPHK1

OAT

TFAP2A

GLS

F2R

CTSL

CNTN2

SIK2

PXN

RAB5A

HSP90B1

TARDBP

AASS

MTHFD1

OPA1

NADK

NAT1

CYP51A1

DUSP4

KCND2

HYOU1

XRCC1

TNFRSF8

CALB1

BBC3

S100A1

BLZF1

DEFB1

TOR1AIP1

GABPB1

ORAI2

INTS10

TOR1AIP2

H2BC21

GGT2

NOTCH3

MAP3K7

PIK3R1

MYD88

TGFB2

SYK

ATP1A1

FLNA

RIPK1

EDNRB

CPT1A

GSN

IL2RB

PPP1CA

PGK1

AKR1C4

C3

GALT

CTNNA1

MTHFR

MUC1

CYP2A6

PFN1

PLD2

SLC4A1

LBR

RUNX2

BECN1

CFI

PTGS1

FCER2

ETFB

DDOST

EIF4G1

S100B

NFATC2

PTPN12

IKBKE

LAT

HSPG2

ITCH

CCNA2

CBR3

BSG

PABPN1

RPL35

S100A4

CBX3

AGPS

CRY1

TJP1

EGR2

VDAC2

BNIP3

HBA2

NDUFA4

SEPHS1

MFN1

CYB5B

COX5B

MPC1

NDUFA5

PABPC1

PLS3

LETM1

VSNL1

CCT2

G3BP1

SMPD2

TAGLN2

HSPA1B

HSPE1

CCN2

ATAD3A

SFXN1

NFYA

TNPO1

RASD1

KCND1

FUBP1

GPX5

SSBP1

MRPL12

MPC2

SEPHS2

CHCHD3

CSN2

FYB1

MRPS14

SQOR

SELENOK

LOC110973015

LOC111365141

ERBB2

PPP3CA

HDAC6

PTPRC

ACVRL1

ACTN1

CAPN1

LPL

LRRK2

HDAC1

RB1

XIAP

B2M

PRKCZ

NTRK1

TPM1

TBP

STIM1

VWF

MCM2

MCM4

ALDH7A1

FPR1

CYP2B6

CYP3A5

POU5F1

SCP2

TPM3

TLR8

RAP1A

TGFB3

VCL

LMNB1

CCR5

ACTN4

NDUFS3

NRG1

HSD17B4

TLR7

MCM3

EZR

BID

ABCC8

CDK9

MYH14

NDUFS1

EMD

HNRNPA1

SERPINH1

ST14

UBE2L3

TMPO

POLG

ADD1

MCM5

CCR6

ANGPT2

ADAR

RNASEH1

RICTOR

CRYAA

SHMT2

PPP3CB

RPTOR

PGRMC1

STMN1

PYGB

LONP1

BMI1

MCM6

GSTA4

PAM

NUP107

IQGAP1

PICALM

KPNB1

KIF5B

ID1

USF1

UBC

VDAC3

GDF2

LTC4S

COPS5

EDEM1

EEF1B2

CDH13

MLST8

NFYC

GZMA

AAAS

MTCH2

GPT

CAPZA2

PTBP1

CAPZB

CCL4

GORASP1

RPL30

PRG2

NFYB

NLN

SSRP1

TPM4

RCN2

PPP1R15A

AGFG1

ACIN1

HOXB5

CORO1C

RHOD

EDF1

DSTN

CCL3

RPL28

PDLIM5

SESN1

PPP1R9B

RRBP1

TMOD3

S100A12

ARPC4

MICAL2

COX7A2

HEXIM1

DDX21

IL27

SAFB

LARP1

STOML2

TWF1

EDC4

ALYREF

HP1BP3

CSTF3

MYO18A

RPL26L1

IFI27

SAP30BP

RALY

TBCA

IL4I1

FUBP3

MRPS18B

P4HTM

INTS8

LRRC59

MICAL3

SEPTIN9

SERBP1

RTCB

PNN

MT1G

CSN1S1

SNU13

ATP5PO

SMCP

RBM27

CGB5

H3C1

FABP12

SEPTIN7

H3C14

H2BC1

GET1

H2AC18

DVL1P1

LOC106736470

CDK4

HDAC4

SLC9A1

ZAP70

IL2RA

PPIB

PTPRF

ACAT1

HCK

F2

ALDOA

AK2

ABCA1

COL2A1

RRM2

PLG

RAB7A

STK4

KRT18

CAMK2G

HADHB

ECHS1

PAX6

KAT2A

YWHAQ

ACO2

DLAT

MYOD1

CDC25B

CD79A

PSAP

PCK2

NAGA

NARS2

S100A6

PPBP

RAPGEF4

PMPCA

TFAP2C

TFE3

VARS2

AKR7A2

ABCB8

GCAT

MIPEP

GLUD2

MARS2

ATP13A2

LTA

FBXW11

FANCF

AK3

BMP3

FKBP8

ACSL5

FOXE1

MAP2

ATXN2

CTSE

HOMER2

CPT1C

CLCN5

HNRNPD

CHIA

CERK

HBG1

MTO1

DPEP1

DUT

MOCS2

ELAC2

NDUFAF4

NDUFB3

IL16

PDSS1

PRRX1

SFRP2

SERPINB3

IDUA

PROCR

SDC3

NME4

PNPT1

LDHC

TREX1

TST

RARS2

KCNMB1

KLK6

KPNA1

RAB11B

SUMO3

TK2

PDCD10

LGMN

EIF2AK1

EARS2

NAGS

DHTKD1

PMPCB

TFB1M

THPO

XRCC2

YME1L1

ADCYAP1

ALOXE3

FLCN

CARS2

CAPN10

GJA3

GLYAT

LTK

BBOX1

APLP2

LTB4R2

ATAD1

ASAH2

ALOX15B

ANO1

AGK

AGPAT5

MCAT

BCAP31

ACOX3

FOLR2

FOXC1

CA3

FCAR

CYB5R1

CYB5R2

CLDN5

GK2

GSTA1

GSTA2

COX4I2

COX8A

HIBADH

CLDN10

HACE1

HCCS

MTFMT

MRPL3

MORF4L1

DECR1

DARS2

RPL3

NDUFA8

NDUFAF1

NDUFB10

NEDD8

RPL34

PFKFB4

PDHA2

SFXN4

SEL1L

SH3KBP1

IFNA2

PARS2

IARS2

PARL

IFITM3

PPM1K

RYR3

SDHAF2

SARS2

RTN4IP1

ENSA

NMNAT2

SLC7A8

SLC25A32

SLC30A5

OPLAH

SLC25A21

NT5C

SEMA3E

SLC22A8

SIL1

SSH1

PSME1

SRM

TOP1MT

TRIT1

TRNT1

TRPC5

TRPM3

REV1

TCP1

KLC1

TERF1

SULT4A1

SUCLG2

KCNE2

TKTL1

KSR1

PRIM1

TP53I3

POLI

KCNK2

ST8SIA2

POLRMT

KIF3A

VAMP8

VRK2

ULK2

WWTR1

LIPT1

MAT2B

GDF11

GNG5

B3GALT4

FBXO32

BACH2

FGL1

APOC1

FGF20

LSM4

AS3MT

ASGR1

ASGR2

ALDH1L2

BPHL

MAVS

ABCB10

ABCD2

ACYP2

ADAMTS17

AADAT

ACSM1

ACP6

FN3K

FOXRED1

C1GALT1

CRYBA1

CROT

GTPBP3

COX7A2L

COX7B

COQ3

HIC1

DNAJA3

DGCR8

DIO1

DIO2

DMRT1

MT1X

DUSP16

GAST

MPP1

DEGS1

NDUFS5

NDUFV3

NDUFAB1

NDUFAF2

NDUFAF3

NDUFB4

NDUFB6

HPR

CD69

GRIN3B

PEX11B

IL27RA

PDLIM1

PDSS2

SFXN5

OXR1

IFRD1

S100P

NHLRC1

NFE2

NLRX1

SLC25A29

PITX3

OS9

HSD17B8

NUDT2

SLC25A14

SLC25A18

NUP153

NT5C3A

PHB2

PIGH

PTPRR

L2HGDH

SYNJ2

TBPL1

TDP2

RECK

STC2

RAB6A

RAB8A

RAB8B

RAD9A

IL32

INF2

IL33

LIMS1

SDSL

S100A7

SNAP91

THG1L

TIMM17A

VRK3

UXS1

LMOD1

GFM2

MGAT5

AZU1

GGT7

MICU1

ARNTL2

MANF

ANKRD26

AIF1

AGMAT

BCL2L13

ME3

ABCC12

ABCG4

AANAT

ACSM3

ACSM5

ACSS1

FASTK

ATP10D

CRLS1

CTF1

CD5L

GRPEL1

COX11

COX20

RHOT2

COQ4

COQ5

CLYBL

HERPUD1

CLPX

CHCHD10

CERKL

MRPL44

MTIF2

MRPS7

MRRF

MTX1

MTX2

ECSIT

NDUFC2

NDUFB2

NEIL1

GPR65

HOGA1

PRX

PELP1

PRSS2

SFXN2

PCBD2

NIF3L1

NIPSNAP1

OPA3

SLC25A31

SLC9A9

HSPB7

PMAIP1

OGDHL

SLC25A36

SLC25A39

SLC25A46

SLC52A3

NUBPL

PGRMC2

PGS1

IMMP2L

LCN1

TRAM1

TNFAIP2

UGT1A7

LDHAL6B

TTC19

TACO1

JPH1

REEP5

TBL2

RDH13

RAB11FIP5

RAB4B

QRSL1

TMEM126A

TMEM126B

PI3

OXSM

TRMU

DNASE2

DUSP13

NCOA4

NDUFA3

CNGA2

SEC14L2

NDUFB7

MT1E

MRPL1

RAB3D

IFI30

ISCA2

THEM4

TIMELESS

ISCU

THBS1

SPTBN1

CEACAM3

RPL14

POLDIP2

PIK3CA

SPTAN1

MYH10

FLNB

ACOX1

NFS1

SLC2A4

GPD1

ELK1

RPS3

GPC1

RPS2

RPS20

RPS26

RPS13

RPS17

RPL31

RPL13

RPS9

NFU1

RPLP0

RPL7

RPS4X

RPL23

RPS25

TCF19

MIR21

MAP2K1

JAK2

CTSD

FYN

PAH

GNAI2

ALDH1A1

ACP5

TRAF6

IRF1

PRKCB

PDP1

ITGAL

TUFM

DNM3

HSD17B1

MT2A

CSF3

BOLA3

BOLA1

MT-CO1

DBH

EIF4E

SERPINE1

MEF2C

YES1

MAP2K6

CYP27A1

LEP

ACADSB

ELANE

TRPV1

GPC4

CTNS

SDC4

TUBB4B

TRPC4

CUL1

GNB4

FANCG

HTT

SDC1

PON2

GLS2

DDB1

SETX

SDCBP

RPS27

RPLP2

RPS15A

SRF

MAFG

AKAP12

MYOF

PPL

MT1A

UTRN

PELO

SNTB2

AQP8

MPRIP

RPL38

COA6

RPL22L1

KBTBD4

DCAF11

ITPRID2

METTL26

PCNA

DHFR

STK11

FGF1

FANCA

CYP3A4

NMNAT1

EIF4EBP1

PINK1

ACADS

TYRP1

DAO

PON1

FOSL1

KNG1

ENDOG

UCP3

SP3

SLC25A10

FLAD1

CYP46A1

CD63

DCT

AGR2

OXT

IAPP

RPS8

YBX1

DDX39A

BAIAP2L1

ENC1

MIR155

STAT1

ADAM10

HGF

TLR2

SLC1A3

PGR

ITGB2

BRCA2

HDAC3

PRKCQ

QDPR

GCDH

GOT1

FANCD2

CYP11B2

MSN

TALDO1

KRT8

GGCX

GPI

SLC40A1

ITGAM

SPP1

AVP

MAP2K7

APOB

ERN1

CXCL12

NDRG1

SAT1

SIRT5

GZMB

GP6

CSF2

CST3

HAMP

GRIN2C

IL18

PLXNB1

KCNN3

ASIC1

TRAF4

SURF1

GJC1

CD163

ADH1C

UBE2E3

GP1BB

AMBP

MTHFD1L

PPIG

LCP1

POLG2

NFIC

HNRNPL

TRIM22

SCG5

IFI6

IPO11

KYAT1

ATP5F1E

PSEN1

RAD51

CDKN2A

DNMT3A

NOTCH1

GSK3B

MME

DNMT3B

ITGB1

JAK1

COL1A1

PRKDC

PHGDH

TRPV4

ASS1

ACTA2

ACACA

IKBKG

IDE

SLC1A2

TRPC6

YY1

GDNF

CCNB1

GJB1

F7

BACE1

EPHX1

HK2

CDH5

NPC1

SLC18A2

TUBB1

IRF3

IRS1

KCNJ5

VAV1

ALAD

CASP4

FKBP1A

BTRC

CNR1

DDX5

HMGCL

NPPA

SLC25A1

ITGAV

AFP

GATM

FH

ARG2

ATF2

ADCY10

CSTB

EIF2S3

SHMT1

S100A10

HSD11B2

TLR9

XBP1

XPC

ALAS1

ARSB

ATP2A3

ATF3

FGF4

ALDH3A1

AGER

FOXM1

MYO5A

DDIT4

TRAF2

UBE2D2

KCNQ4

WNT2

AKR1B10

BDH1

GHRL

EWSR1

ATG7

MGST1

C5AR1

LOXL1

COX10

CPA6

PPOX

LCN2

ASPH

ATP8A1

ADAM8

COMMD1

CETN2

IL17A

SLC30A8

SRI

RAD23A

SELL

CLCN3

DNASE1

RPL19

PER1

NPC2

IFNB1

ICMT

RPL4

TTPA

UBIAD1

RERE

PXDN

PRIM2

UTS2

MAFF

ATF7

ALDH1L1

FLVCR1

CXCL2

DHX9

CD93

DDX17

MOCS1

PDLIM7

PRDM2

PLEK

SLC30A1

LDHD

S100A2

ABCF2

HEPH

RFK

SLPI

SLC39A1

TOMM20

UBE2E2

KPNA6

FTMT

HNRNPH1

CETN1

CIAPIN1

NOXA1

KLHL41

PAM16

CYP20A1

TIMM10

GADD45GIP1

COX16

H2AX

EDEM3

EFS

MRPL41

VEGFD

MELTF

SLIRP

TRA

COX17P1

MTCO2P12

TRC-GCA24-1

CDK6

AKT3

CDK2

AXL

HK1

HDAC2

TGFBR2

GCK

CACNA1H

CBL

DNMT1

SPARC

TNFRSF10B

CASP2

MAP2K3

GRIN1

RXRA

SLC6A3

TTR

TUBB3

LDLR

LIMK1

LDHA

UBE2I

CFTR

DUOX1

KDR

NAMPT

NQO2

CYP21A2

EPO

LOC107372315

CXCR4

ITGB3

SCO1

CA9

AGTR1

CA2

ITGA4

BCAT1

INTS6

RAC2

TXNDC15

NHLRC2

VASN

GRXCR1

DNAJC16

LDHB

CCL11

MTRR

MSRA

SMG1

COQ9

IDH2

TH

FASLG

ANXA5

HLA-B

LOX

HPX

IDH1

COMT

KCNMA1

CD36

HSP90AA1

F3

EIF2S1

CCL5

NRF1

FOSB

PGAM1

AHCY

IGF1

SDHB

HBB

COX4I1

DCXR

AKR1C1

ME1

GSTM2

DNAJA1

AHSP

IL6

MDM2

TLR4

HNF4A

CYP1A1

EPHA3

RELB

TOR1A

EEF1A1

RNASE1

CYP2D6

UCHL1

CASP7

VIM

TNFRSF1A

SLC16A1

AKR1C3

PDK1

CUL3

PARP2

ANGPT1

HNF1A

HMGCR

CEBPB

SECISBP2

TRDN

ROMO1

FTH1

CYP11A1

FDXR

COX17

ABCG2

NPM1

PRKCA

AKR1C2

TNFSF10

CFLAR

ACHE

DUSP3

KCNA5

XRCC6

SLC23A2

PTGIS

MFN2

APAF1

CD81

DUSP1

TRPA1

HSPA6

CTTN

PPIF

SAMHD1

HSPA1A

ETHE1

NOX3

TOMM40

ERP29

ABL1

SLC2A1

MDH2

KDM1A

LMNA

DPYSL2

STAT5A

ELN

SERPINB2

MTTP

NUMA1

TRAP1

BANF1

ID3

DEK

CLEC4A

IKBKB

MYB

ANXA1

SMAD3

PLD1

ABCC2

PIN1

CYP2C19

ETFA

DHRS4

TPH1

TPR

EPX

HAGH

RPA3

PTPRU

SUOX

PRKAB1

BCR

ABCB1

MMP3

PLAU

RARA

PRMT1

SET

PIK3CB

VHL

BAD

REL

CD38

PTPRA

TP73

CR2

OCLN

CPOX

PTPRS

TRIM28

STIP1

CNBP

CTRL

PTPN11

MYC

FTL

CYGB

SELE

CREBBP

SCN5A

MAPK12

TAP1

CDK1

FBP2

IL18R1

SNCB

TRADD

SLC3A2

RBX1

PSMD10

ACBD3

NOP58

MRTFA

EP300

ANXA2

HSPD1

TNFRSF1B

ADAM17

SPR

MAP3K1

CYP2E1

CALM1

JUND

DHDH

INTS4

GSTT1

CAV1

GCH1

ACADM

POU2F1

NR2E3

ATP5F1A

RET

FLT3

TOP2A

YWHAE

RRM2B

CACNA1C

MAPK9

DES

YWHAZ

GFAP

FADD

BDNF

KCNB1

HLA-A

MOG

PSMD4

PSMD14

ELAVL1

PSMC3

PSME3

BCLAF1

GOLGA3

KRAS

ITGA2B

GLUL

FASN

ACE

TRPC3

MAP2K4

CRP

UCP2

CS

S100A8

AIFM2

INTS5

GJA1

MAPT

CTH

PRKAA2

FGF2

PGD

BLVRA

CYP1A2

SLC25A3

IL2

SELP

PPARA

MBP

HAAO

TUBA1B

MTF1

MAFK

DNAH8

COQ10A

SELENOM

METAP2

SLC1A5

INTS9

LYN

TSC2

ACTB

PSEN2

CYP2C9

NR3C2

TPO

NME1

TNFSF11

TNFRSF11B

PPARGC1A

MTHFD2

NUDT1

RPL10A

SELENOP

PRKCD

ACTG1

PRKCE

APOA1

CYP1B1

CSK

TSC1

TGM2

IL10

PLCG1

PRODH

ITGA2

HSP90AB1

SLC25A5

ALOX12

DUOX2

E2F1

MYO1C

PEX5

PLEC

ITIH4

TG

MSRB2

JUNB

TGFB1I1

MYL12A

DBN1

GSX1

ALDH2

PTPN6

ETS1

ESR2

PLA2G4A

RHEB

NR1H2

EIF2AK3

CD40LG

PPIA

JUP

CLU

HDAC9

ADIPOQ

ALOX15

PDGFA

UCP1

TSPO

FGF7

PTPA

ATP5F1C

CTNNB1

NFKB2

MMP14

IRAK1

DSP

SERPINA1

GATA4

EEF2

UGT1A1

KAT2B

FANCC

CHAT

SLC1A1

GNAI1

CBR1

FECH

SCARB1

RHOB

DAXX

SULT1E1

IL5

ARF4

LTF

CRYZ

SKP1

RPL27

SSR4

LPA

RPL12

RPL13A

MYO1D

PLXNC1

RCC2

LIMA1

MYO1B

NOC2L

INTS7

TMEM37

DNM2

PIK3R2

PLAT

KCNQ1

WT1

CASP6

CACNA1B

FGG

ANPEP

ADRB2

ACE2

CYP2C8

CDKN1B

GRIN2D

CALCR

PRKCI

PDPK1

NR2F2

HTR2C

NOD2

OPRM1

SLC12A6

SPTLC2

TNNI3

KCNJ2

KCNN4

IL6R

RUNX1

POMC

VRK1

CARM1

CAPN2

GK

CCR3

MAP4K4

LRP1

ANTXR2

ATP1B1

ERCC2

ADORA2B

ADSL

ACTA1

FOLR1

CTSH

CDKN1C

HADH

DIAPH1

CD28

DDX58

COL3A1

CYSLTR2

CSF3R

CD79B

RHO

PDHX

PPP2R1B

HTRA2

PRKACB

PRKACG

PAX3

SOX2

SCN1A

SCN2A

SCN3A

SCN8A

SCN9A

SCNN1A

SLC6A4

SMAD2

HSPA9

HSD17B3

UBE2D3

UBE3A

ITPA

CYP24A1

CYP27B1

POLB

PLA2G6

OTC

PPARD

KRT5

WNT3A

LIPE

GAMT

GLP1R

AQP2

ETV1

ALDH6A1

BIRC3

ABCB6

C5

CEL

DMD

GPT2

GATA1

NDUFS7

NEDD4L

NEFL

NR4A2

NME2

HSD3B2

SLC25A12

SLC12A5

SLC22A5

TPT1

TYMP

LIG1

KYNU

KAT5

SUCLA2

PYGM

IL5RA

ALAS2

CAMKK2

GNMT

GJA8

F8

FANCL

LRP2

FHL1

MAF

ATIC

MAD1L1

AMACR

AMT

ALDH1A3

ALDH3A2

ADRB3

AGXT

ERG

MECP2

ABAT

ABCC3

ACAD8

ACAT2

MGLL

C1S

ATP2C1

CTLA4

ERCC6

CLCN2

MMAB

CHRNA1

ROS1

E2F4

DDB2

NDUFS8

NDUFV1

PDK4

SERPINI1

OXCT1

PISD

IGFBP3

PCBD1

PCCA

PRKG2

PRF1

RPS6KA5

NGFR

PLA2G2A

PML

SLC19A1

SEMA3A

INPP5D

PTPRB

PHYH

TIMP3

LARS2

TUBA4A

ULK1

ITGB5

STT3A

TCF3

TCIRG1

IVD

SUCLG1

PRKD2

TRPM4

MYH6

RAC3

HTR1B

PCCB

ITPR2

ITPR3

TIMP1

VAPB

LMX1B

ADIPOR1

AFG3L2

GFM1

FA2H

ANXA4

FARS2

ASPA

FBLN5

ALDH1B1

MCCC1

MCCC2

ME2

MECR

FKBP4

BCKDHA

ACSL1

FOXC2

GAL

G6PC

MAD2L2

CX3CR1

CXCL10

CYP2J2

HPSE

CTBP2

APOA2

GPR37

HK3

COX6A1

CLPP

HMBS

CKM

CHMP2B

MYO5B

CD9

CASQ2

CXCR3

NDUFB9

EHHADH

EIF4A1

NDUFA6

NEU1

SKP2

SERPINF1

SERPINF2

IDI1

SOCS3

PAX7

SCN4A

NLRP12

RPS14

SLC7A5

PNPLA2

HSD17B2

SLC25A15

NTHL1

SLC12A7

NPPB

TOLLIP

UGT2B7

LGALS3

LIG3

ITGAX

STS

SYN1

KHK

REV3L

KMO

TCN2

RBP4

KL

KIF1B

SULT2A1

RALBP1

SREBF1

DUSP10

MTHFS

EIF2AK4

NDUFA12

NDUFA13

HMGCS2

CFL2

CUL5

RNASEL

RAPGEF3

TFR2

USP10

YARS2

VAMP1

LITAF

ADIPOR2

ACSF3

ALPI

CAV3

GPAM

GMPPB

BAG1

AUH

MAP1LC3A

ARRB1

APOC3

ARHGAP1

LRPAP1

EPM2A

MBTPS1

MBTPS2

MCEE

MCFD2

BCS1L

MDK

ACAA2

ACAD9

ACADL

GALC

CA5A

FPGS

CXADR

CXCL1

CYP4B1

APOD

EXO1

CD47

RHOQ

RHOT1

COX6B1

HNF4G

CMPK1

HIBCH

CLPB

HFE

CKMT2

MLYCD

CGA

HARS2

MRPS16

MSMO1

MSRB3

MYBPC1

EBP

RPIA

CD86

GRK4

GRK5

SPG7

PDK2

PER2

SI

SFRP4

SFTPB

HTR2B

POU2F2

PALB2

PANK2

IGFBP5

RUNX3

NME3

SLC5A3

SLC7A1

SMAD7

HSD17B7

HSD3B1

PLK2

SLC30A2

SLC25A19

SLC25A24

SLC12A4

SLC22A4

SLC13A3

PTN

PIAS4

PSMD9

PHOX2B

TRAF3IP2

LAP3

UGCG

TRIB3

TRPM1

TAC1

SYP

RETN

TCL1A

TARS2

ING1

P2RX2

PDYN

SMN1

POLR2C

NUDC

PSMC5

PSMD11

PSMD12

PICK1

PTPRM

UBE2K

UBE2S

RASSF2

KIDINS220

RAB1A

PTX3

RAB5C

TLN1

PSMD3

PSMB6

DCTN2

CIB1

EBF1

RANGAP1

RAB1B

GOLGA2

CBX1

BAG2

AP3D1

AP3S1

FARSA

AMOT

AK4

ERLIN2

BPTF

MDC1

ACTR1A

CPVL

AP1M1

HDLBP

DNAJA2

EEF1E1

EEF1G

EFHC1

MOB1A

EIF3H

NOP10

PQBP1

SMARCA5

PITPNB

POLR2H

NUP93

NXF1

PHF6

SRP72

PSMC6

PSMD1

PSMC2

TPD52

UFM1

KCNS3

TAX1BP1

RCC1

RBM4

RBM8A

STX5

DHX30

PPA1

RBBP7

ZC3H14

CACYBP

MAGED2

ADRM1

AHSA1

AASDHPPT

ACTL6A

CXCL14

CYP2W1

HPS5

CPSF6

COPZ1

HNRNPDL

HNRNPH3

CGN

CHMP2A

MTA2

NAP1L1

NAP1L4

DDX23

DDX39B

EIF3C

EIF3D

EIF3I

RPL32

COPE

IFNA1

SNX2

SAP18

SAV1

RPL36

NFKBIE

INA

PSMD13

PTMA

TPSB2

SYMPK

STOX1

RAB9A

STK26

NCBP1

OTUB1

POLR3K

PIP4K2C

IRS4

WTAP

GEMIN4

LUC7L

FIP1L1

AGTRAP

ACOT8

HNRNPAB

RNPS1

EIF3B

EIF3E

EIF3G

IK

PAG1

RSL1D1

NOLC1

SLMAP

ILF2

RCVRN

PSMD6

TP53INP1

MYCBP

DSC1

EIF3K

SCAMP3

TIGAR

USO1

VAT1

FLG2

GATAD2A

CCT8

MARCKSL1

BASP1

API5

ACOT9

CBLC

CAPRIN1

CDK11A

COPS7B

COPS8

CKAP4

CHMP4A

MLEC

DNAJB5

DERL2

DHX8

NAA50

CD2BP2

MOB1B

DAZAP1

NDFIP2

ELP2

HNRNPUL1

SPIN1

PRPF19

SERPINB10

NTPCR

SMPD4

PLRG1

NUDT21

SRP19

SRP68

PTMS

SRRT

IST1

STRAP

SUPT16H

TMOD2

DYNC1LI1

DCTPP1

GZMK

EIF3M

POLDIP3

RAB21

THOC1

THRAP3

ZC3HAV1

GAR1

CCAR2

CCDC80

EPRS1

EPDR1

ERH

AARS1

CUTA

CRNKL1

CPSF7

CLCC1

COLGALT1

CHORDC1

MTPN

DNAJC9

DARS1

DCD

COPG1

SERPINB12

SEC16A

SAP30L

SARNP

PHF5A

PSPC1

LARS1

TNRC6C

UPP2

TSPAN4

TTC4

SURF6

TMF1

TSPAN32

NCOA5

SCYL2

TIMM13

WBP11

YTHDC1

EXOSC6

MACROD1

DDX19A

SEC22B

PDCL3

SAFB2

RRP12

PHRF1

UBAP2L

LDHAL6A

TTBK1

KCTD12

RALYL

RBM23

TMED9

TMED4

SF3B6

HTN3

IARS1

COPS4

H3-3A

HRNR

PRPF38A

NOC3L

QPCTL

ZNF32

FOXN2

CWC15

CWC22

MMGT1

CHTOP

H3-3B

MOB2

DPY19L1

HNRNPUL2

SFSWAP

INIP

RABL6

SEPTIN2

ATAD3C

LSM12

FAM98B

AKAP17A

CENPV

CLDN17

H3-4

H3C2

HNRNPA1L2

RBMXL2

TMEM109

ZNF512

CCDC124

H1-0

H3C3

H3C4

KPRP

CAAP1

MACROH2A2

H2BC4

H2BC5

H3C10

H3C12

KNOP1

PBDC1

H1-10

HDGFL2

H2AZ2

H2BC10

H2BC6

H3C11

H3C6

H3C7

H3C8

H2AC7

PPIAL4A

RTRAF

H2AC8

H2BC11

H2BC7

H2BC8

H3C15

POLR1G

H3C13

QARS1

SPINDOC

PALM2AKAP2

MIR200C

H2AC19

PEDS1-UBE2V1

MIR661

MIR1246

LOC111589215

DDC

CASP1

MGMT

DGKE

SOS1

IL4R

PTPN22

TGFA

ZBTB16

ANG

NR4A3

HTR3A

PDE2A

P2RX7

THBS2

ADH4

HIPK2

USP2

MYBL2

GRK1

GPD1L

BHLHE40

BST1

CDH23

METAP1

HLF

PSIP1

SLC24A2

LMF1

KCNMB3

TMPRSS11A

AASDH

ZACN

CCN1

MIR328

CYP19A1

OGDH

SIRT6

RAF1

CFL1

TAT

TUBB

NLRP3

RPS6

PRKN

IL1R1

AOX1

S100A9

RPL27A

CYB561

SESN2

PRKAG1

PHB

MAP3K14

HP

MTFP1

DNAJC4

MRPL33

MRPL52

MRPS36

CCDC51

CYSTM1

SERP1

IFNA14

IFNA16

PRELID2

SMDT1

SLN

PLGRKT

SLC25A34

SLC25A48

TMEM143

TRIM4

LGALS7B

TTC23

TAMM41

TMEM242

TMEM134

PSTK

COX8C

TIMM21

ZCCHC24

ZNF580

ZBED5

ATP5PF

CYB561D1

RIDA

MRPL58

MT-ND4

MT-ND5

DUSP28

NAXD

CCDC58

MRM2

SDHAF3

TMEM223

PRR5L

NME1-NME2

KYAT3

ARMS2

ATP5PB

ATP5MG

MTARC2

DEFB4B

MRM3

ELSPBP1

NIPSNAP2

NRDC

AKR1B15

FAM210A

ATP5MC1

ATP5MC2

ATP5ME

MRPL57

CCDC167

TOMM70

TEX19

TMEM256

ANP32C

FDX2

H19

MTERF2

MT-ND4L

MTARC1

DELE1

OXLD1

SLX4IP

INTS11

AFG1L

CEMIP2

COA8

CFAP74

DGLUCY

DMAC2L

SERPINA2

PRELID3A

SDHAF4

SSC4D

MICOS10

TCAIM

MAIP1

BICDL1

FMC1

ETFRF1

HDHD5

MTRES1

DEPP1

DMAC2

PRELID3B

PEDS1

RAB5IF

MIR126

MIR27A

FAM215A

H2BS1

TMEM35B

MIR141

MIR34B

MIR34C

ANXA2P2

MIR122

RIPOR3

MIRLET7A1

MIR17

MIR200B

MIR206

MIR93

MIR342

AFG3L1P

MIR378A

GGT3P

MIR590

MIR455

PCGEM1

MIR1-2

MIR30D

MIR520D

MIR675

LINC01619

MIR15A

SNORA12

DUX4L1

VTRNA1-1

VTRNA1-2

ISCA1P1

FAM136BP

AOX3P

TXNP1

GLRXP2

TXNP5

TXNP6

GLRXP1

TXNP2

TXNP4

CHCHD2P8

LOC727947

HBB-LCR

MSBP1

TXNP3

HDL3

LOC171417

LOC117134604

LOC117134605

LOC117134606

LOC117134607

LOC117134608

LOC117134611

LOC117135104

LOC117135105

LOC117135106

LOC110599569

AASDH

AASDHPPT

ABHD10

ACACA

ACACB

ADH4

AGT

AIFM1

AKR1A1

ALKBH1

ALS1

APEX1

ARMD1

ARNTL

ATG4A

ATORS

ATPIF1

BLVRA

BMI1

BRAF

CARD19

CBS

CCS

CGDX

CHCHD4

CISD1

CISD2

CISD3

CLOCK

CMTX4

CNR1

COQ2

COQ6

COX20

COX7C

COXPD29

CPT1A

CTBP1

CTBP2

CYB561A3

CYBB

CYGB

CYP17A1

DDIT3

DEE82

DEFB1

DJ1

DNMT3A

DNMT3B

DUSP1

EI24

ENC1

ENOX1

ERAF

ERO1L

ERO1LB

ERVW1

FAM213A

FAM49B

FECH

FLAD1

FOXO3A

GAPDH

GCLM

GDPD5

GLRX2

GOT1

GOT2

GPD1

GPD2

GPX4

GRPEL1

GRPEL2

GSK3B

GSR

H6PD

HAAO

HSD11B1

HYOU1

IDH2

IMDDHH

ITPR1

KEAP1

KYNU

LCN2

LYN

MAP3K5

MFM8

MICAL1

MIEN1

MIR1-1

MTCO1

MTCO2

MTCO3

MTCYB

MTF1

NADSYN1

NAPRT

NDUFA12

NDUFS4

NEDMCMS

NFE2L2

NFYC

NMNAT1

NMNAT2

NMNAT3

NNMT

NNT

NOS1

NOS3

NOX1

NPAS2

NQO1

NQO2

NUDT1

NXN

P4HB

PBD1A

PDHA1

PDILT

PDK1

PDK2

PDK3

PDK4

PDSS1

PGAM5

PKM

PNKD

POR

PRDX1

PRDX2

PRDX4

PRDX5

PRKAA1

PRKG1

PTGES

PTPN1

PTPN11

PYROXD1

RAC1

RNF7

RNH1

ROMO1

RSMD1

RYR1

SCARA3

SCO1

SCO2

SELENON

SELENOP

SELENOT

SELENOW

SENP1

SESN1

SESN2

SFRP2

SH3BGRL2

SH3BGRL3

SHC1

SHPK

SLC25A30

SLC2A10

SLC2A4

SLE

SOD1

SRXN1

SUMF1

TIMELESS

TMTC1

TMTC2

TMX2

TMX3

TMX4

TP53

TP53I3

TRPC1

TRPC5

TRPM2

TXN

TXN2

TXNDC12

TXNDC17

TXNDC2

TXNDC4

TXNDC8

TXNIP

TXNL1

TXNRD1

TXNRD2

TXNRD3

UCP1

UCP2

VCRL1

VCRL2

BCL2A1

BNIP3L

CAPN3

GIT1

GPHN

LGALS13

MAGT1

MEIS1

MIR6855

NEWENTRY

SCRIB

SH3BGRL2

SNAI2

STEAP3

TTN-AS1

USP17L9P

WNT5A
